# Annual variation in breeding success in boreal forest grouse: Four decades of monitoring reveals bottom‐up drivers to be more important than predation

**DOI:** 10.1002/ece3.9327

**Published:** 2022-10-09

**Authors:** Per Wegge, Robert Moss, Jørund Rolstad

**Affiliations:** ^1^ Faculty of Environmental Sciences and Natural Resource Management Norwegian University of Life Sciences Ås Norway; ^2^ Station House Crathes, Banchory, Kincardineshire UK; ^3^ Department of Forest Genetics and Biodiversity Norwegian Institute of Bioeconomy Research Ås Norway

**Keywords:** bird breeding success, boreal forest, grouse, hypothesis testing, information theory, NAO, path analysis, *Tetrao*

## Abstract

Knowledge of the temporal variation in reproductive success and its key driving factors is crucial in predicting animal population persistence. Few studies have examined the effects of a range of explanatory factors operating simultaneously on the same population over a long period. Based on 41 years of monitoring (1979–2019), we tested prevailing hypotheses about drivers of annual variation in breeding success in two sympatric species of boreal forest grouse—the capercaillie (*Tetrao urogallus*) and the black grouse (*T. tetrix*)—in a 45 km^2^ boreal forest landscape. From counts in early August, we measured breeding success (chicks/hen) along with potential determining factors. We formulated five main hypotheses on causes of variation (hen condition, chick weather, chick food, predation, demographic characteristics) and derived 13 associated explanatory variables for analysis. We first tested the five hypotheses separately and then used model selection (AICc) to rank the best predictive models irrespective of hypotheses. Lastly, we used path analysis to illuminate potential causal relationships. Barring demographic characteristics, all hypotheses were supported, most strongly for chick food and predation. Among predictor variables, chick food (insect larvae and bilberry fruit crops), vole and fox abundances, the winter‐NAO index, and temperature after hatching, had the strongest effect sizes in both species. Precipitation after hatching had no detectable effect. Model selection indicated bottom‐up factors to be more important than predation, but confounding complicated interpretation. Path analysis suggested that the high explanatory power of bilberry fruiting was due not only to its direct positive effect on chick food quality but also to an indirect positive effect on vole abundance, which buffers predation. The two components of breeding success—proportion of hens with broods and number of chicks per brood—were uncorrelated, the former having the strongest effect. The two components had different ecological correlates that often varied asynchronously, resulting in overall breeding success fluctuating around low to moderate levels. Our study highlights the complexity of key explanatory drivers and the importance of considering multiple hypotheses of breeding success. Although chick food appeared to equal or surpass predation in explaining the annual variation in breeding success, predation may still be the overall limiting factor. Comparative and experimental studies of confounded variables (bilberry fruiting, voles, and larvae) are needed to disentangle causes of variation in breeding success of boreal forest grouse.

## Graphical abstract

Bottom‐up and top‐down drivers keep density‐independent reproductive success at low to moderate levels in two ground‐nesting boreal forest grouse. Four decades of monitoring reveals bottom‐up drivers to be more important than predation. Our study highlights the complexity of key explanatory drivers and the importance of considering multiple hypotheses of breeding success.

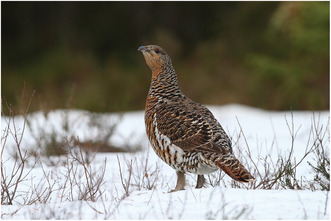

## 1 INTRODUCTION

Boreal forest grouse (*Tetraonidae*) has declined through most of Western Europe (Storch, 2007) during recent decades, commonly explained by poorer breeding success (Jahren et al., 2016). Traditionally, population regulation has been discussed in the context of density dependence: as density increases, factors that depress vital rates become successively stronger, eventually stabilizing population size to fluctuate around an equilibrium level (Lack, 1954; Turchin, 1999; Wolff, 1997). Krebs (2002) argued that in order to solve the ongoing rather fruitless debate about density‐dependent regulation, research should have a “mechanistic” approach, whereby effects of explanatory factors on vital rates are more thoroughly examined. Our long‐term study is an attempt to do so. Aiming to identify the main drivers of annual variation in reproduction, we examine how breeding success varied with a range of bottom‐up and top‐down factors in two species of Eurasian boreal forest grouse, the capercaillie (*Tetrao urogallus*) and the black grouse (*T. tetrix*).

Some long‐term studies have examined aspects of breeding in birds (e.g., song sparrows (Arcese et al., 1992); Seychelle warblers (Brouwer et al., 2012); acorn woodpeckers (Koenig et al., 2011); white‐throated dippers (Nilsson et al., 2011); eiders (Coulson, 2010; Morelli et al., 2021)), but causes of variation in avian breeding success have largely been inferred from short‐term, single‐factor correlations. In Eurasian boreal forest grouse, no previous study has looked at several potential factors operating simultaneously on the same population over a long period. Here, we examine several prevailing hypotheses, and their associated explanatory variables, based on quantitative measurements of environmental and demographic factors thought to influence breeding success of capercaillie and black grouse. Data were collected on sympatric populations of the two species in a boreal forest landscape in southeast Norway over a period of 41 years (1979–2019). In order to identify the main drivers, we first tested the predictions of each main hypothesis, after which we compared the best explanatory variables from each hypothesis by means of information‐theoretical model selection (AICc) and path analysis.

### 1.1 The hypotheses

Capercaillie and black grouse are large (hens weigh about 2.0 and 0.8 kg, respectively), ground‐nesting birds, widely distributed across the extensive Eurasian boreal biome. Characteristic features of their breeding phenology include mating at leks in early spring, clutches of 7–9 eggs laid by well‐camouflaged hens in well separated ground nests and incubated for 3.5–4 weeks. The hens alone rear broods of precocial chicks that feed largely on insect larvae for their first 3–4 weeks and fledge to independence 3 months after hatching. During this long period of incubation and chick rearing, many environmental factors may influence the number of chicks reared to independence. From research on breeding ecology and population dynamics of boreal grouse, we formulated five main hypotheses (some including sub‐hypotheses), deduced associated predictions, and selected potential explanatory variables to test the predictions (Table 1).

**TABLE 1:** Response and explanatory variables used for testing hypotheses about drivers of breeding success in capercaillie and black grouse at Varald State Forest during 1979–2019.

Main hypotheses	Sub‐hypotheses	Variable	Years sampled	Characteristics	Predicted direction of response in breeding success	Observed response in breeding success *β* (SE)

*Response variables*				

Breeding success	41	Number of chicks per hen		

Brood frequency	41	Number of broods per hen		

Brood size	41	Number of chicks per hen with brood		

*Detrending variable*				

NHT	41	Northern Hemisphere Temperature	**+**	**0.30** (0.11)

*Explanatory variables*				
Hen Condition		NAO_w_	41	Winter NAO (DJFM)	**–**	**−0.34** (0.10)

T_8wPre_	41	Temperature 8 weeks before hatching	**+**	0.10 (0.11)

SnowFree	41	Date of snow free ground (80% snow free ground)	**−**	0.12 (0.11)
Chick Weather		T_4wPost_	41	Average daily min. Temperature 4 weeks after hatching	**+**	**0.32** (0.12)

P_4wPost_	41	Amount of rain 4 weeks after hatching	**−**	−0.03 (0.11)
Chick Food	Food Quantity	Larvae	28	Abundance of insect larvae after hatching	**+**	**0.43** (0.12)

Plant Stress	BB_(*t*−1)_	41	Abundance of bilberry fruits in August of year *t*−1	**+**	**0.40** (0.10)

Plant Stress	T_JJA(*t*−12)_	41	Mean JJA temperature during years *t*−1 and *t*−2	**−**	0.18 (0.13)
Predation	Alternative Prey (APH)	Voles	41	Abundance of bank and field voles in August	**+**	**0.39** (0.10)

Red Fox	Foxes	41	Abundance of red foxes in winter prior to grouse census	**–**	**−0.32** (0.11)

Delayed Raptor	Grouse_(*t*−23)_	38	Mean density of grouse during late summers *t*−2 and *t*−3	**–**	**−0.20** (0.10)
Demography	Age‐dependence	YoungHen	40	Proportion of juvenile hens in breeding hen population	**–**	0.24 (0.12)

Density‐dependence	DensHen	41	Density of hens in breeding hen population	**–**	0.15 (0.12)

*Note:* Standardized regression coefficients (*β* ± SE) of explanatory variables with breeding success (capercaillie and black grouse combined) are shown partialled out (detrended) with Northern Hemisphere Temperature (NHT). Coefficients significant at one‐tailed *p* < .05 in predicted direction are boldfaced.

#### 1.1.1 Hen condition hypothesis

Capercaillie and black grouse evolved in northern boreal forests with a continental climate characterized by cold winters and dry snow. During winter, hens subsist mainly on low‐quality, arboreal foods, and conserve energy by roosting in snow burrows whenever possible. The species mate in early spring when the ground is partly covered with snow. At this time, hens must rebuild their body reserves for laying eggs and for the energy‐draining incubation period. Access to sprouting new ground vegetation is critical to their nutritional status. Poor condition or nutritional stress in this early period hampers breeding performance via low‐quality eggs and reduced viability of chicks. It also forces hens to leave their nest more often, or to pursue longer feeding bouts during incubation, thereby exposing themselves and their eggs to predation (e.g., Brittas, 1988; Gregg & Crawford, 2009). We therefore hypothesized that cold, dry winters and early, warm springs should benefit hen condition and hence breeding success.

Large‐scale climate indices sometimes predict ecological processes better than local weather statistics (Hallett et al., 2004). One such is the North Atlantic Oscillation (NAO), the difference in sea‐level atmospheric pressure between the Azores and Iceland. This strongly influences winter climate over western Europe (Hurrell et al., 2003) and to a lesser extent in summer (Folland et al., 2009). A positive winter (DJFM) index leads to stronger westerly winds that transport warm, moist oceanic air toward Scandinavia, providing mild, wet, and windy winter conditions. By contrast, when the index is negative, westerlies are suppressed and northern Europe experiences cold, dry, and calm winters. European grouse studies using the NAO index to characterize the effects of winter and summer weather on breeding success have provided mixed evidence (black grouse, Barnagaud et al., 2011; willow ptarmigan (*Lagopus lagopus*), Kvasnes et al., 2014; red grouse (*L. lagopus scoticus*), Vergara et al., 2012).

Effects of hen condition on egg quality and breeding success have received support from indirect evidence on several grouse species (Moss & Watson, 1984; Swenson et al., 1994; Zwickel & Bendell, 1967), plus strong empirical evidence on sage grouse (*Centrocercus urophasianus*) in USA (Gregg, 2006) and lapwing (*Vanellus vanellus*) in Sweden (Blomqvist et al., 1997).

*The Hen Condition Hypothesis predicts* that breeding success should benefit from cold winter weather with dry snow, and hence a negative winter NAO index, and that it also should benefit from warm weather and early snow‐free ground before mating.

#### 1.1.2 Chick Weather Hypothesis

Newly hatched grouse chicks thermoregulate poorly (Marcström, 1960) and depend on warmth from the brooding hen. During their first 3–4 days, a yolk sac provides supplemental nourishment that helps regulate body temperature—independent self‐regulation is achieved when the chicks are about a week old. For the next month or so, they need especially nutritious food such as arthropods for rapid growth. During cold or wet weather, feeding bouts become interrupted by needed brooding, which slows growth and weakens the chicks' physical condition—so making them more susceptible to starvation and predation. This hypothesis has been supported in several studies (e.g., Ellison & Magnani, 1984; Erikstad & Spidsø, 1982; Marcström, 1960; Moss, 1986; Moss et al., 2001; Watson & Moss, 2008).

*The Chick Weather Hypothesis predicts* that breeding success should benefit from warm weather and suffer from precipitation following hatching.

#### 1.1.3 Chick Food Hypothesis

The Chick Food Hypothesis comprised two sub‐hypotheses—one quantitative and one qualitative. The Food Quantity sub‐hypothesis involves larvae of butterflies, moths, and sawflies (Lepidoptera and Hymenoptera) feeding on new leaves of bilberry (*Vaccinium myrtillus*)—a crucial, protein‐rich food for chicks during their first few weeks (Picozzi et al., 1999; Savory, 1989; Spidsø & Stuen, 1988; Wegge & Kastdalen, 2008). The abundance of larvae is known to fluctuate markedly between years, presumably due to weather (Reynolds et al., 2007). The idea that the quantity of larval food available to chicks should influence breeding success has previously been supported by both direct (Picozzi et al., 1999) and indirect evidence (Atlegrim & Sjöberg, 1995; Baines et al., 2017).

The qualitative Plant Stress sub‐hypothesis stems from White (1984, 1993), who proposed that stressed plants reduce their chemical defenses and so become more susceptible to herbivory. This prompted Selås (1997) to put forward “the mast depression hypothesis,” whereby high crops of bilberry fruit reduce chemical defense compounds in bilberry vegetation, making it more digestible for voles and grouse chicks in the following year. Selås, Sonerud, et al. (2011) later added that cold summer weather during a masting year and the year before should accentuate the stress on the plants, making them even more nutritious for voles and grouse in the post‐masting year. Selås (2000) presented a positive correlation between bilberry fruit production and capercaillie abundance—but not breeding success—in autumn of the following year.

*The Chick Food Hypothesis predicts* that breeding success should be positively influenced by (1) abundance of larvae on bilberry plants after the chicks hatch (food quantity), and (2) bilberry fruit production the previous summer, along with a negative influence of summer temperature in the previous 2 years (food quality).

#### 1.1.4 Predation Hypothesis

The Predation Hypothesis involved three sub‐hypotheses. First, according to the Alternative Prey sub‐hypothesis (APH: Angelstam et al., 1984; Hagen, 1952; Lack, 1954), predation on nests, chicks, and adult grouse should vary inversely with the abundance of fluctuating voles. When voles (primary prey) are sparse, generalist mammalian predators—mainly red fox (*Vulpes vulpes*) and pine marten (*Martes martes*)—should prey more heavily on alternative prey (grouse and mountain hares [*Lepus timidus*]), thereby increasing the mortality of these alternatives. This sub‐hypothesis has received much support in Scandinavia (Angelstam et al., 1984; Breisjøberget et al., 2018; Hörnfeldt, 1978; Marcström et al., 1988; Steen et al., 1988; Wegge & Storaas, 1990), but less so in Finland (Lindén, 1988; Lindström et al., 1995). A corollary of APH is that the red fox should raise more offspring when its main prey—voles—is abundant (Lindström, 1988). Hence, we expected the growth rate (*λ*) of foxes to track vole abundance, thereby influencing grouse breeding success via a numerical effect (see below) in addition to the functional effect of a dietary shift.

Second, since red fox and pine marten are main predators of boreal forest grouse (Baines et al., 2016; Kauhala et al., 2000; Kurki et al., 1997; Lindström et al., 1994; Marcström et al., 1988), the breeding success of grouse should decrease with increasing densities of these two mesopredators. This sub‐hypothesis has not been examined independently of the APH sub‐hypothesis (above). We did not have reliable field data on marten abundance, and so our hypothesis refers only to the abundance of red fox—henceforth the Red Fox sub‐hypothesis.

Third, Tornberg et al. (2005) predicted that grouse chicks should suffer higher mortality from increased numbers of breeding goshawks (*Accipiter gentilis*) following peak densities of the main goshawk prey species (grouse, hares, and squirrels [*Sciurus vulgaris*])—the Delayed Raptor sub‐hypothesis. Goshawks typically do not breed until 2–3 years of age (Krüger, 2005), so this effect on grouse should be delayed by 2–3 years. We did not have reliable census data on breeding goshawks and so used total grouse density—a major prey group according to this hypothesis—as a surrogate for goshawks. Except for some indirect evidence (Selås & Kålås, 2007; Tornberg et al., 2012), the Delayed Raptor sub‐hypothesis has not yet been explicitly tested.

*The Predation Hypothesis predicts* that breeding success should (1) fluctuate synchronously with the abundance of voles, while varying inversely with (2) the abundance of red fox and (3) the autumn density of grouse 2 and 3 years earlier. It also predicts that the growth rate of red fox should track vole numbers.

#### 1.1.5 Demography Hypothesis

The Demography Hypothesis comprised two sub‐hypotheses. First, juvenile grouse hens typically rear fewer chicks than older hens, apparently investing less in breeding because clutch sizes are smaller and, in large grouse species, some juveniles do not breed at all (Zwickel & Bendell, 2004). Hence, breeding success varies with the age composition of breeding hens in spring—the Age‐dependence sub‐hypothesis (Lindström et al., 1997). A large data set on black grouse reproduction has supported this hypothesis (Willebrand, 1992).

Second, in dense populations, individuals compete for resources to get access to optimum feeding and nesting sites. This depresses their physical condition and might also depress their breeding performance—the Density‐dependence sub‐hypothesis (Blomberg et al., 2017; Lindström et al., 1997). Although several studies have examined the potential role of density‐dependence in avian breeding success (e.g., Lack, 1966; Sæther et al., 2016), no such study has been reported on boreal forest grouse.

*The Demography Hypothesis predicts* that breeding success should vary inversely with (1) the proportion of young hens and (2) the density of hens in spring.

## 2 MATERIALS AND METHODS

### 2.1 Study area

**FIGURE 1:**
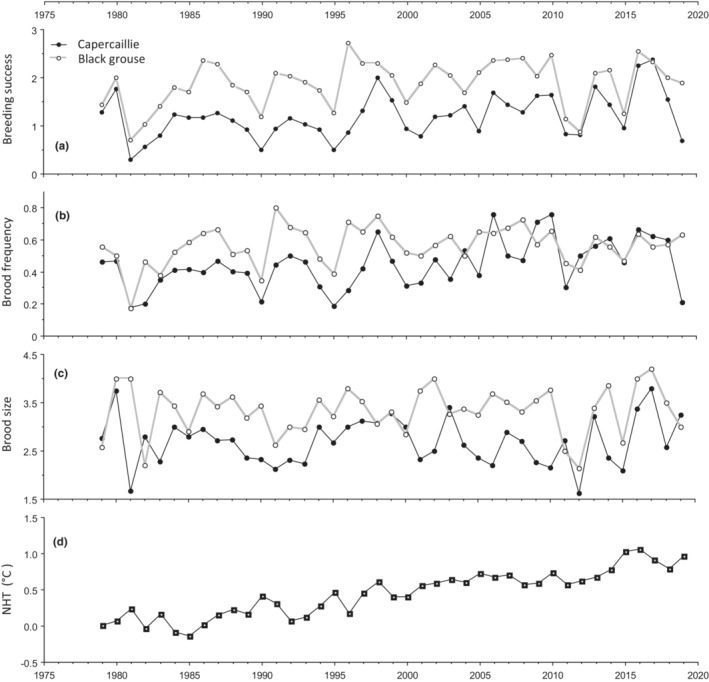
Time series of (a) breeding success (ratio of chicks to hens), (b) brood frequency (proportion of hens with brood), and (c) brood size (ratio of chicks to hens with brood) in capercaillie and black grouse at Varald State Forest, southeastern Norway, during 1979–2019. (d) The Northern Hemisphere Temperature (NHT, HadCRUT4nh) is shown, as it was used as a detrending variable throughout the analyses.

The study was conducted at Varald State Forest, next to the Swedish border in southeast Norway (60°10′ N, 12°30′ E; Appendix S1: Figure 1). The terrain is gently undulating between 200 and 400 m a.s.l., comprising Norway spruce (*Picea abies*) and Scots pine (*Pinus sylvestris*) interspersed with scattered birch (*Betula* spp.) and aspen (*Populus tremula*). Climate is transitional between coastal and continental, with mean summer (JJA) and winter (DJF) temperatures of 15 and –6°C, respectively. From late November to late April, the ground is usually covered with snow. During the course of the study, increasing temperature led to shorter winters and earlier springs (Wegge & Rolstad, 2017). Average summer (JJA) precipitation was 250 mm.

The 45‐km^2^ study area is contiguous with other mixed conifer forests on all sides, interspersed only with a few small, peripheral patches of abandoned farmland. Timber has been harvested for centuries, since the late 1940s by clearcutting. During the course of this study, the area of old semi‐natural forest was gradually reduced from 50% to 20%, with spring densities of capercaillie and black grouse fluctuating around estimated averages of 2.0 and 2.7 birds per km^2^, respectively (P. Wegge, unpublished material). There is a dense population of moose (*Alces alces*), fewer roe deer (*Capreolus capreolus*), and semi‐resident wolves (*Canis lupus*) and lynx (*Lynx lyn*x). Fluctuating numbers of stoat (*Mustela erminea*), weasel (*M. nivalis*), pine marten, badger (*Meles meles*), and a dense population of red fox are the main predators of small mammals and ground‐nesting birds. Main raptors are common buzzard (*Buteo buteo*) and goshawk. Quasicyclic voles—*Microtus agrestis* and *Myodes glareolus*—are common in grassy and bilberry‐dominated habitats, respectively. Earlier studies have identified fox and marten as the main predators of grouse nests and chicks (Wegge & Rolstad, 2011).

### 2.2 Data collection

**FIGURE 2:**
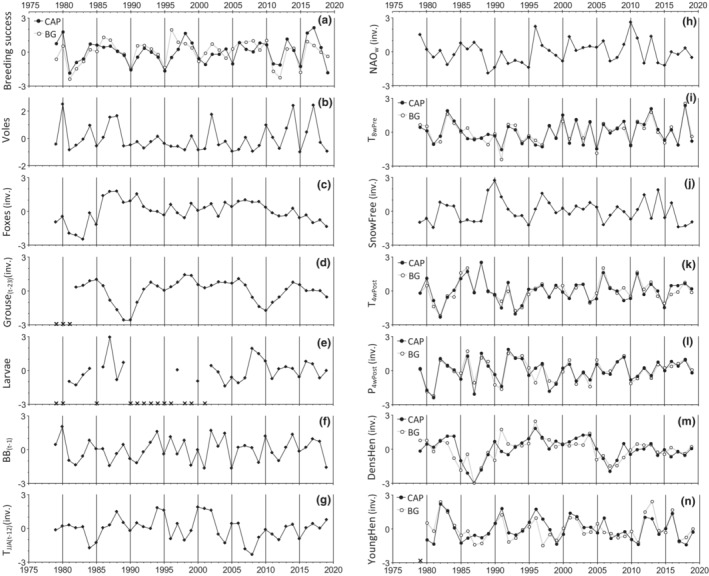
Time series (1979–2019) of breeding success (a) and the 13 explanatory variables (b–n) that were used in the analyses. All variables are detrended with The Northern Hemisphere Temperature (NHT), standardized to Z‐scores (zero mean divided by 1 SD), and presented in the predicted direction, i.e., variables with negative effects are inverted (inv.). Years with no data are denoted with *x*. CAP, capercaillie; BG, black grouse.

Demographic data were obtained by counting birds in 22 c. 2 km^2^ blocks during August using trained pointing dogs (number of blocks, census effort, and sample sizes are given in Appendix S1: Figure 2). Flushed, fully grown birds were classified to species and sex, while the number of chicks in their broods was counted. Numbers observed per 10 h of sampling provided indices of density, which for hens was used as a surrogate for breeding density in spring—reasonable because intervening summer mortality of hens has been negligible in the study area (Wegge & Rolstad, 2011). The proportion of juvenile hens in spring was estimated from the proportion of female chicks in the previous August count (Appendix S1: Sampling). Any emigration of dispersing female chicks during autumn and early spring was assumed to be balanced by immigrants prior to breeding, as our study area was contiguous with similar habitats outside it. Dates of peak mating and subsequent hatching were estimated from direct observations at leks, supplemented by monitoring 4–6 leks with remote cameras since 2015.

To test the Hen Condition and the Chick Weather Hypotheses, we downloaded local weather statistics from Kongsvinger meteorological station, at 150 m elevation 25 km from the study area. Initially, we assembled 31 weather variables, reducing these to five during preliminary analyses (Appendix S1: Weather, and Appendix S2: Table 1). Temperatures and snow depth were adjusted for differences in elevations (Wegge & Rolstad, 2017). We used winter NAO as a regional index of winter weather. Spring and summer NAO indices did not correlate at all with breeding success, nor did they explain any significant aspects of the local spring and winter weather data. Thus, they were not included in the final analyses. We used the Northern Hemisphere Temperature Index (NHT, Appendix S1: Weather) to account for a long‐term increasing trend in temperature (see Section 2.3.3 and Figure 1d).

For the Predation Hypothesis, we sampled voles in late August–early September by baited snap traps along six transects in grassy and bilberry‐dominated habitats. Yearly abundance indices, calculated for each habitat type separately, were expressed as the number of voles captured per 100 trap‐nights. Unless specified, the variable Voles is the mean of the two indices (Appendix S1: Predation). Red fox abundance was estimated from different sources: counting of tracks in winter along forest roads and fixed snowmobile routes and ski tracks (19 years, >60 km/year), and local and regional hunting statistics (1979–2019; Appendix S1: Predation).

For the Chick Food Hypothesis, we sampled larvae in late May and June by sweep netting at 6–10 fixed stations in bilberry‐rich sites within old spruce‐dominated stands. Lepidoptera and Hymenoptera larvae were counted and grouped into three size classes: small (<5 mm in length), medium (5–12 mm), and large (>12 mm). The larval index was expressed as numbers of large and medium sized larvae per 10 sweeps. We assessed the abundance available to chicks by interpolating the indices to 8–10 days after hatching (Appendix S1: Chick food). During the last 17 years, we counted the number of bilberry fruits (berries) in randomly distributed circular plots within bilberry‐rich, old forest at fixed sites in August. After correcting for ramet coverage, berry abundance was indexed as numbers per 1 m^2^ of bilberry plants. We also had access to bilberry fruit indices in the study area for the whole period of 41 years, based on newspaper records (Selås et al., 2021). Parallel analyses for the final 17 years, using either our field measurements or the newspaper index, gave very similar results (Appendix S1: Chick food)—we therefore used the latter here.

### 2.3 Statistical analyses

Counts in August included the number of hens with or without broods plus the number of chicks in each brood, which were well‐grown at the time of the counts. Our primary measure of breeding success, for each species separately, was the ratio of chicks to hens. This could be broken down into the proportion of hens with brood (brood frequency) multiplied by the ratio of chicks to hens with brood (brood size). In regression models, we use these ratios as response variables, rather than the number of chicks (or broods) with the number of hens (or broods) as offset: the latter would have favored years with larger sample sizes and also put more weight on the more numerous black grouse. There were no correlations between census effort and breeding success, brood frequency, or brood size (Appendix S1: Sampling, and Appendix S1: Figure 2).

#### 2.3.1 Modeling approach

First, we tested predictions from each hypothesis separately by means of linear multiple regressions for breeding success or its components (brood frequency and brood size) as response variable and measures of each hypothesized cause as explanatory variables. The two grouse species were treated as subjects in a repeated measures analysis of deviance via SAS (ver. 9.1) Proc Mixed, specifying “species nested in year” in the REPEATED statement and “variance components” as the covariance structure. In further analyses, we investigated possible differences between species by including interactions between them (categorical) and explanatory (continuous) variables. Proc Mixed fits models via REML (Restricted Maximum Likelihood) assuming that the response variables, but not necessarily the explanatory variables, are normally distributed. Accordingly, we checked for each species that the distributions of breeding success and its two components did not depart significantly from normal. Response and explanatory variables were standardized to Z‐scores (subtracting the mean and dividing by its standard deviation) to facilitate direct comparisons of effect sizes. Predictions from each hypothesis were directional, and so we denote test results with one‐sided type‐I error rates <0.05 as statistically significant. Non‐directional hypotheses (e.g., possible differences between species) were tested two‐sided. If not otherwise stated, we denote effect sizes *β* of 0.20–0.25 as weak, 0.25–0.35 as moderate, and ≥0.35 as strong. Throughout we use effect size in the statistical sense of the slope of the regression coefficient, which does not necessarily imply biological causation.

Second, we ranked the various hypotheses by combining their standardized slopes, adding those in the hypothesized direction and subtracting the very few that were not. This produced a composite “explanatory value” for each hypothesis. Standard deviations were combined via simulation: from each of the normal distributions described by an estimate and its standard error we picked a random sample and added these together to get an estimated explanatory value. We repeated this 10,000 times and calculated the standard deviation of the 10,000 estimates.

Third, we sought the best predictive models for breeding success, brood frequency, and brood size, respectively, irrespective of hypotheses. Candidate models combined the best explanatory variables from each hypothesis, added in order of their slopes as discovered during hypothesis testing. We ranked models within a group according to AICc (Burnham & Anderson, 2002) and selected the most parsimonious model (ΔAICc ≤2 for each extra parameter) while excluding “uninformative parameters” (Arnold, 2010). Confounding among explanatory variables from different hypotheses complicated the straightforward application of statistical criteria to model selection. Therefore, in order to disentangle direct from indirect causal relationships, we did path analyses (Blums et al., 2002; Mitchell, 1992) for each species separately via the SAS Calis procedure. Path analysis required Pearson correlation coefficients as inputs, and these had to be estimated from normally distributed pairs of variables. We therefore took natural logarithms of the three variables that included a few abnormally large values: voles (adding 0.1 because there were some zeros), foxes, and larvae. This resulted in path coefficients that were smaller than comparable standardized regression coefficients calculated during hypothesis testing. As an informal aid to assessing the relative strength of path coefficients, we estimated (by simulation) conditional one‐tailed probabilities *p*_*c*_ that the normal distribution described by each coefficient and its standard error would include zero or a more extreme value.

#### 2.3.2 Choice of variables

Initially we collected 45 possible explanatory variables of which weather data (31) were most numerous (Appendix S2: Table S1). Some variables were essential to a particular hypothesis, and these were retained for further analysis. There were also subsets of similar variables that represented alternative versions of the same biological phenomenon (e.g., temperature for 2 or 4 weeks after hatching). From each such subset we retained variables that correlated well with breeding success in both species. The final filter involved combining retained variables in linear regressions that represented alternative versions of each hypothesis and examining their partial slopes (*β*‐coefficients) and influence on model AICc scores. The 13 variables finally retained to represent each hypothesis are presented in Figure 2, Table 1, and Appendix S2: Table 2.

#### 2.3.3 Trends and autocorrelations

Two response variables—breeding success and brood frequency—displayed long‐term positive trends, especially in capercaillie. Such long‐term trends were also present in some explanatory variables, most pronounced for summer temperature. As we were interested in teasing out year‐to‐year variation, we looked for potential detrending variables. Preliminary analyses showed that Northern Hemisphere Temperature (NHT) and a linear trend based on successive years gave very similar results when used as alternatives. As they confounded each other when entered into the same model, we chose to use NHT as a detrending variable because it also represented gradually increasing temperature due to global warming (Figure 1d), thereby having physical and biological meaning.

When testing the APH, we detected evidence of a 3–4‐year cycle in vole abundance. This raised the specter that correlations between vole abundance and grouse breeding success could be due to similar vole and grouse cycles coinciding by chance. However, the evidence for a similar cycle in grouse breeding success was negligibly small. In addition, APH regressions using AR1 residuals were virtually indistinguishable from ones using the original variables.

Another possible joint autocorrelation structure for response and explanatory variables occurred in the Demography Hypothesis. Here, breeding success in year *t* − 1 was used to calculate the explanatory variable YoungHen for year *t*. Another difficulty arose because breeding success in year *t* − 1 might have influenced the other explanatory variable DensHen in year *t*. In each case, models substituting AR1 residuals for the original variables led to the same conclusions—both types of models are reported.

## 3 RESULTS

### 3.1 Breeding success, brood frequency, and brood size

**FIGURE 3:**
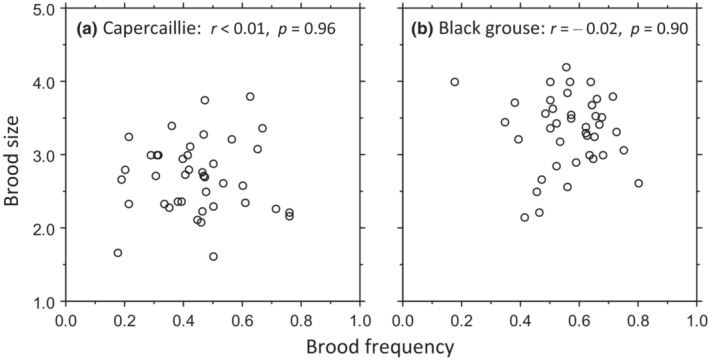
Correlations between brood frequency (proportion of hens with brood) and brood size (ratio of chicks to hens with brood) in (a) capercaillie and (b) black grouse.

During the 41‐year study, the breeding success of the two species fluctuated synchronously in a quasiperiodic pattern (Figure 1). Black grouse had higher proportions of hens with broods and also larger brood sizes, resulting in their average overall breeding success being 55% higher (1.9) than that of capercaillie (1.2) (Table 2a). In each species, brood frequency and brood size correlated strongly with breeding success—notably, brood frequency explained about twice as much of the variation in breeding success as brood size (Appendix S3: Figure 1a–d). Surprisingly, and importantly, there was no correlation between brood frequency and brood size—they varied completely independently of each other in each species (Figure 3, Appendix S3: Figure 1e–f). Thus, high values of each rarely coincided, resulting in breeding success fluctuating around low to moderate levels (Figure 1a). Along with the synchronous fluctuation, breeding success in the two species was highly correlated (*r* = 0.67), with brood frequencies correlating more strongly (*r* = 0.48) than brood sizes (*r* = 0.38; Appendix S3: Figure 1g–i). This strong synchronicity allowed us to combine the species in most analyses, an approach also consistent with the fact that none of the hypotheses distinguished between species.

**TABLE 2:** (a) Mean breeding success, brood frequency, and brood size of capercaillie and black grouse over 41 years at Varald State Forest (1979–2019). (b) Comparison between and within species of total loss, loss due to complete loss of clutches/broods (i.e., mostly nest loss), loss due to chick mortality in broods with ≥1 chick, and chick mortality sequentially to loss of clutches/broods.

	Capercaillie	Black grouse	Difference between species	
			*t*	*p*
**(a) Components of breeding success**				
Breeding success (chicks/hen)	1.20 (0.072)	1.88 (0.075)	−11.35	<.001
Brood frequency (broods/hen)	0.44 (0.024)	0.57 (0.019)	−5.47	<.001
Brood size (chicks/brood)	2.69 (0.077)	3.34 (0.078)	−6.78	<.001
**(b) Loss %**				
Total loss^a^	83.1 (1.0)	77.0 (0.9)	7.66	<.001
(i) Complete loss of clutch/broods^b^	55.6 (2.4)	43.5 (1.9)	5.47	<.001
(ii) Chick loss in broods ≥1 chick^c^	62.1 (1.1)	59.3 (0.9)	2.20	.034
(iii) Chick loss in broods ≥1 chick sequential to complete loss^d^	27.5 (1.6)	33.5 (1.2)	−3.47	.001
**Difference within species**				
(i) vs. (ii)				
*t*	−2.51	−7.43		
*p*	.016	<.001		
(i) vs. (iii)				
*t*	7.20	3.29		
*p*	<.001	.002		

^a^ (i) + (iii).

^b^ (1 – brood frequency) × 100.

^c^ ([Clutch size – brood size]/clutch size) × 100.

^d^ (Brood frequency × (ii)) × 100.

Losses are compared as percentages based on average clutch sizes of 7.1 in capercaillie and 8.2 in black grouse (see Supporting Information, Appendix S1: Sampling, for details). Two‐tailed tests of differences in mean values with SEs in brackets

Brood frequency was determined by hens losing entire clutches or broods, and so we could infer the relative importance of complete loss of clutches and whole broods versus partial loss of chicks within hatched broods. The former predominantly represents nest loss and the latter chick mortality. Complete loss of clutches (and broods) contributed significantly more to the total loss than the partial loss of chicks within broods, especially in capercaillie (56 vs 28%) but also in black grouse (44 vs 34%; Table 2b), which is consistent with the finding that brood frequency was better than brood size at explaining variation in breeding success (Appendix S3: Figure 1a–d). Because brood frequency and brood size were uncorrelated, models were tested with each as separate response variables.

### 3.2 Testing the main hypotheses

#### 3.2.1 Hen condition

**FIGURE 4:**
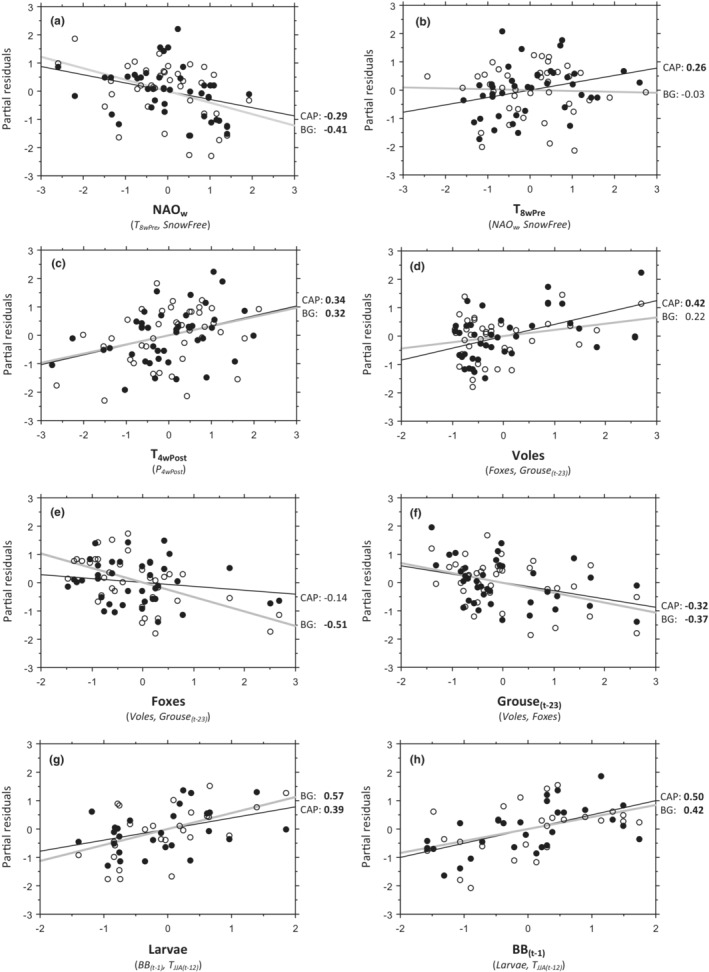
Partial residual plots showing the relationships between breeding success and explanatory variables given that other independent variables are controlled for in the model. Capercaillie (CAP: ●) and black grouse (BG: ○). Variables controlled for are given in brackets. The Northern Hemisphere Temperature (NHT) is included as detrending covariate in all models. Estimated *β*‐coefficients are shown to the right, with those significant at *p* < .05 (one‐tailed type I‐errors in predicted direction) boldfaced. See Table 3 and Supporting Information (Appendix S5: Table 1) for standard errors and more details and differences between species.

None of the local winter weather variables (temperature, precipitation, and snow depth) was useful in explaining variations in breeding success (Appendix S4: Table 1a). Even so, breeding success was clearly and negatively associated with winter NAO (Figure 4a), mostly via brood frequency (Table 3). This was quite surprising, because NAO correlated rather strongly with all three local winter weather variables: high NAO values were associated with mild and wet winters with little snow (Appendix S4: Table 1b). In capercaillie, but not in black grouse, warmer spring weather during the 8 weeks before hatching affected breeding success positively (Figure 4b), solely via a moderate effect on brood frequency (Appendix S5: Table 1). There was no discernible effect on breeding success of the date in spring when ground became snow‐free (Table 3). Spring and summer NAO indices did not explain any variation in breeding success.

**TABLE 3:** Effects on breeding success of the explanatory variables presented as standardized partial *β*‐coefficients (slopes) with SEs from multiple regressions within each main hypothesis.

Hypothesis	Sub‐hypothesis	Years of data used in models	Explanatory variable	Breeding success	Brood frequency	Brood size
				*β* (SE)	*β* (SE)	*β* (SE)
**Hen condition**		41	NAO_w_	**−0.35** (0.11)	**−0.36** (0.11)	−0.10 (0.11)
		41	T_8wPre_	0.11 (0.10)	0.13 (0.11)	−0.01 (0.11)
		41	SnowFree	−0.00 (0.11)	−0.10 (0.11)	0.16 (0.12)
**Chick weather**		41	T_4wPost_	**0.32** (0.12)	0.18 (0.12)	**0.31** (0.12)
		41	P_4wPost_	0.01 (0.10)	−0.07 (0.11)	0.08 (0.11)
**Chick food**	Food quantity	28	Larvae	**0.48** (0.11)	**0.41** (0.12)	**0.24** (0.14)
	Plant stress	28	BB_(*t*−1)_	**0.46** (0.12)	**0.30** (0.12)	**0.36** (0.14)
		28	T_JJA(*t*−12)_	−0.11 (0.13)	0.03 (0.13)	**−0.27** (0.16)
**Predation**	Alternative prey (APH)	38	Voles	**0.32** (0.10)	0.09 (0.11)	**0.40** (0.11)
	Red fox	38	Foxes	**−0.33** (0.11)	**−0.37** (0.12)	−0.05 (0.12)
	Delayed raptor	38	Grouse_(*t*−23)_	**−0.34** (0.10)	**−0.27** (0.11)	**−0.23** (0.11)
**Demography**	Age‐dependence	40	YoungHen	0.20 (0.12)	0.12 (0.13)	0.21 (0.13)
	Density‐dependence	40	DensHen	0.05 (0.12)	0.10 (0.12)	−0.09 (0.12)
**Demography ‐ AR1**	Age‐dependence	39	YoungHen	−0.02 (0.13)	−0.09 (0.13)	0.28 (0.13)
	Density‐dependence	39	DensHen	0.06 (0.15)	0.09 (0.15)	−0.05 (0.15)

*Note:* Models include data from capercaillie and black grouse combined. Statistical significance, at one‐tailed *p* < .05 in predicted direction, is indicated with boldface. (See Figure 4 and Appendix S5: Table 1 for differences between species).

#### 3.2.2 Chick weather

Warm weather during the 4 weeks following hatching had a moderately positive effect on breeding success, mostly mediated through an effect on brood size (Table 3, Figure 4c). Surprisingly, we found no effect of precipitation on any aspect of breeding success, neither of the total amount of rain nor of the frequency of days with rain during 4 weeks after hatching (Table 3, Appendix S5: Table 1). We also checked whether breeding success was related to rainfall only during colder weather. However, this was not confirmed, as there was no difference between the birds' response to rainfall at high and low temperatures (species combined, *F* < 0.01, *p* = .98), nor was there any difference between the two species in this response (*F* = 0.28, *p* = .60; Appendix S4: Figure 1, and Appendix S4: Table 2). Thus, rainfall had no effect on breeding success irrespective of ambient temperature.

#### 3.2.3 Chick food

Tests of the chick food sub‐hypotheses showed that the abundance of insect larvae influenced breeding success strongly and positively (Figure 4g). In the Plant Stress sub‐hypothesis, the effect of bilberry fruiting the previous year was strongly positive (Table 3, Figure 4h), affecting both brood frequency (mostly in capercaillie) and brood size (mostly in black grouse; Appendix S5: Table 1). The effect of previous summer temperature was also in the predicted negative direction, although rather weak, and significant only for brood size (Table 3). Analyses of confounding variables modified these results, see section 3.3 below.

#### 3.2.4 Predation

Among the three predation sub‐hypotheses, the abundance of voles—a buffer against predation in the APH—had a moderately positive effect on breeding success (Table 3, Figure 4d). It was mainly mediated through a strong effect on brood size, especially in black grouse (Appendix S5: Table 1). As expected, the growth rate of red fox tracked vole abundance (ln Fox_*λ*_ vs ln Voles_[*t*−1]_: *r* = 0.43, *t* = 2.95, *p* = .005). Secondly, the overall abundance of red fox had a moderately negative effect on breeding success, primarily affecting brood frequency (Figure 4e, Table 3, and Appendix S5: Table 1). Thirdly, the density of grouse 2–3 years earlier—a surrogate for the breeding density of goshawks—had a moderately negative effect on breeding success (Table 3, Figure 4f), affecting both the frequency and the size of broods (Appendix S5: Table 1). Analyses of confounding variables modified these results, see 3.3 below.

#### 3.2.5 Demography

Contrary to predictions, breeding success tended to increase both with the proportion of young hens and with the total density of hens in spring. Table 3 presents two versions of the demography model: the first uses original variables, the second uses AR1 residuals to remove a possible effect of joint autocorrelation structure in response and explanatory variables (see section 2.3.3). In any case, these results clearly refute the two predictions from the Demography Hypothesis.

#### 3.2.6 Ranking of main hypotheses

Aiming to rank the hypotheses in importance, the “explanatory value” of each hypothesis comprised the accumulated partial regression slopes of the relevant variables (Table 4). The Chick Food and Predation Hypotheses explained breeding success 3–6 times better than the Chick Weather and Hen Condition Hypotheses. In capercaillie, this was mainly due to brood frequency, for which the Hen Condition hypothesis also attained a high value, whereas in black grouse, it was mainly due to brood size.

**TABLE 4:** The Composite Explanatory Values with SEs of each main hypothesis for breeding success, brood frequency, and brood size.

Main hypothesis	Explanatory variables	Breeding success			Brood frequency		Brood size	
		Combined	CAP	BG	CAP	BG	CAP	BG
Hen condition	NAO_w_, T_8wPre_, SnowFree	0.14 (0.18)	0.26 (0.24)	0.01 (0.25)	**0.61** (0.22)	−0.06 (0.28)	−0.45 (0.28)	0.03 (0.29)
Chick weather	T_4wPost,_ P_4wPost_	0.28 (0.24)	0.28 (0.30)	0.29 (0.32)	0.08 (0.28)	0.17 (0.29)	0.33 (0.34)	0.27 (0.35)
Chick food	Larvae, BB_(*t*−1)_, T_JJA(*t*−12)_	**0.96** (0.23)	**0.86** (0.30)	**1.07** (0.29)	**0.71** (0.29)	0.40 (0.29)	0.37 (0.35)	**1.40** (0.35)
Predation	Voles, Foxes, Grouse_(*t*−23)_	**0.84** (0.21)	**0.71** (0.29)	**0.96** (0.28)	**0.59** (0.27)	0.48 (0.27)	0.34 (0.33)	**1.04** (0.34)

*Note:* Variables within each main hypothesis are listed in descending order of their contribution. Significant values at one‐tailed *p* < .05 in predicted positive direction is boldfaced. Values are from the 27 years when all variables were recorded. (See section 2.3.1 for definition of Explanatory Value).

### 3.3 Combining hypotheses

#### 3.3.1 Model selection via AICc

Our attempt to construct a set of predictive models, irrespective of hypotheses about biological causation, was bedeviled by confounding between variables—especially from the Chick Food and Predation Hypotheses. Thus, the model that best predicted breeding success (lowest AICc) included larval abundance, bilberry fruit production the previous year, winter NAO, temperature after hatching, and density of grouse 2–3 years before (Table 5a, model 1). However, model 5 (Larvae, BB_[*t*−1]_ and NAO_w_) ranked as the best after rewarding parsimony and excluding uninformative parameters. The two most influential variables were larvae (*β* ~ 0.50) and bilberry (*β* ~ 0.35) from the Chick Food Hypothesis. Surprisingly, none of the seven best predictive models included voles or foxes from the Predation Hypothesis, although these had been strongly supported when this hypothesis was tested separately. Even so, foxes attained statistical significance in models 8 and 10 with a parameter estimate of −0.19.

**TABLE 5:** Candidate models listed in order of AICc, combining the most influential variables affecting breeding success, brood frequency, and brood size respectively from the four main hypotheses of Hen Condition, Chick Weather, Chick Food, and Predation. Estimated slope parameters are based on data from capercaillie and black grouse combined for the 27 years with records of all variables.

Models^a^								AIC_c_	∆AIC_c_	LL	*K*	*w*
**(a) Breeding success**												
(1)	**0.51** Larvae	+**0.34** BB_(*t*−1)_	−**0.24** NAO_w_	+**0.24** T_4wPost_	−**0.27** Grouse_(*t*−23)_			123.4	0.0	49.7	8	0.43
(2)	**0.48** Larvae	+**0.37** BB_(*t*−1)_	−**0.28** NAO_w_		−**0.23** Grouse_(*t*−23)_			125.4	2.0	52.8	7	0.16
(3)	**0.53** Larvae	+**0.34** BB_(*t*−1)_		+**0.29** T_4wPost_	−**0.27** Grouse_(*t*−23)_			126.0	2.6	53.1	7	0.12
(4)	**0.40** Larvae	+**0.37** BB_(*t*−1)_	−**0.23** NAO_w_	+**0.20** T_4wPost_				126.1	2.7	53.1	7	0.11
(5)	**0.39** Larvae	+**0.40** BB_(*t*−1)_	−**0.27** NAO_w_					126.6	3.2	55.2	6	0.09
(6)	**0.43** Larvae	+**0.38** BB_(*t*−1)_		+**0.25** T_4wPost_				128.1	4.7	56.0	6	0.04
(7)	**0.50** Larvae	+**0.38** BB_(*t*−1)_			−**0.21** Grouse_(*t*−23)_			130.0	6.6	56.9	6	0.02
(8)	**0.35** Larvae	+**0.36** BB_(*t*−1)_				−**0.19** Foxes		130.2	6.8	57.0	6	0.01
(9)	**0.42** Larvae	+**0.41** BB_(*t*−1)_						130.4	7.0	58.8	5	0.01
(10)	**0.30** Larvae	+**0.27** BB_(*t*−1)_				−**0.19** Foxes	+0.16 Voles	130.8	7.4	55.5	7	0.01
**(b) Brood frequency**												
(1)	**0.25** Larvae	+**0.18** BB_(*t*−1)_	−**0.23** NAO_w_			−**0.22** Foxes		123.6	0.0	51.9	7	0.22
(2)	**0.22** Larvae		−**0.23** NAO_w_			−**0.26** Foxes		123.7	0.1	53.8	6	0.21
(3)	**0.32** Larvae	+**0.23** BB_(*t*−1)_	−**0.27** NAO_w_					125.2	1.6	54.5	6	0.10
(4)			−**0.24** NAO_w_			−**0.33** Foxes		125.4	1.8	56.3	5	0.09
(5)	**0.23** Larvae					−**0.31** Foxes		125.9	2.3	56.5	5	0.07
(6)	**0.25** Larvae	+0.18 BB_(*t*−1)_				−**0.27** Foxes		125.9	2.3	54.9	6	0.07
(7)	**0.29** Larvae				−0.17 Grouse_(*t*−23)_	−**0.33** Foxes		126.3	2.7	55.1	6	0.05
(8)		+0.14 BB_(*t*−1)_	−**0.24** NAO_w_			−**0.30** Foxes		126.4	2.8	55.1	6	0.05
(9)	**0.35** Larvae	+**0.22** BB_(*t*−1)_	−**0.28** NAO_w_		−0.10 Grouse_(*t*−23)_			126.9	3.3	53.5	7	0.04
(10)						−**0.38** Foxes		127.6	4.0	58.9	4	0.03
(11)	**0.24** Larvae			+0.10 T_8wPre_		−**0.32** Foxes		127.6	4.0	55.7	6	0.03
(12)	**0.27** Larvae	0.20 BB_(*t*−1)_				−**0.26** Foxes	+0.04 Voles	128.2	4.6	54.2	7	0.02
**(c) Brood size**												
(1)				0.20 T_4wPost_			+**0.37** Voles	147.1	0.0	68.7	4	0.35
(2)							+**0.42** Voles	147.2	0.1	70.1	3	0.34
(3)		0.14 BB_(*t*−1)_					+**0.35** Voles	148.3	1.2	69.3	4	0.19
(4)	0.05 Larvae						+**0.41** Voles	149.3	2.2	69.8	4	0.12

^a^ All models of breeding success and brood frequency were detrended with Northern Hemisphere Temperature (NHT).

Significant *β* (one‐tailed *p* < .05 in predicted direction) are boldfaced. LL, log likelihood; K, number of estimated parameters; w, Akaike weight

After rewarding parsimony, the best model for brood frequency included winter NAO and foxes (Table 5b, model 4). The best model for brood size included voles (*β* ~ 0.40) and temperature after hatching (*β* ~ 0.20). However, temperature did not attain statistical significance and was excluded as an uninformative parameter, leaving voles as the sole explanatory variable (Table 5c).

Serious confounding occurred among the bilberry and larvae variables from the Chick Food Hypothesis and voles from the Predation Hypothesis. A fairly strong effect of voles on breeding success (*β* = 0.39) when tested separately was weakened to insignificance (*β* = 0.16) when tested in concert with larvae and bilberry (Table 6). Conversely, when bilberry was combined in a model with larvae, confounding strengthened both effects. This confounding went along with voles being positively correlated with bilberry (significant) and larvae (not significant), while bilberry and larvae were uncorrelated.

**TABLE 6:** Confounding of key variables in the Chick Food and Predation hypotheses explaining breeding success, shown as percent change in slopes of partial regressions when confounding variables are included in the models.

Focal variable	Covariables		Focal variable *β* (SE)	*df*	*t*	*p*	% change in *β*
Voles			0.39 (0.11)	51	3.56	<.001	–
Voles	Larvae		0.31 (0.11)	50	2.87	.003	−21
Voles	BB_(*t*−1)_		0.30 (0.12)	50	2.43	.010	−23
Voles	Larvae	BB_(*t*−1)_	0.16 (0.12)	49	1.28	.103	−59
Larvae			0.39 (0.12)	51	3.26	.001	–
Larvae	Voles		0.29 (0.12)	50	2.52	.008	−26
Larvae	BB_(*t*−1)_		0.42 (0.11)	50	3.89	<.001	+8
Larvae	Voles	BB_(*t*−1)_	0.36 (0.12)	49	3.17	.002	−8
BB_(*t*−1)_			0.37 (0.13)	51	2.93	.003	–
BB_(*t*−1)_	Voles		0.21 (0.14)	50	1.52	.067	−43
BB_(*t*−1)_	Larvae		0.41 (0.11)	50	3.61	<.001	+11
BB_(*t*−1)_	Voles	Larvae	0.32 (0.13)	49	2.41	.010	−14

Models are based on both species combined, and *p*‐values are shown for one‐tailed significance test in predicted direction

#### 3.3.2 Path analysis

**FIGURE 5:**
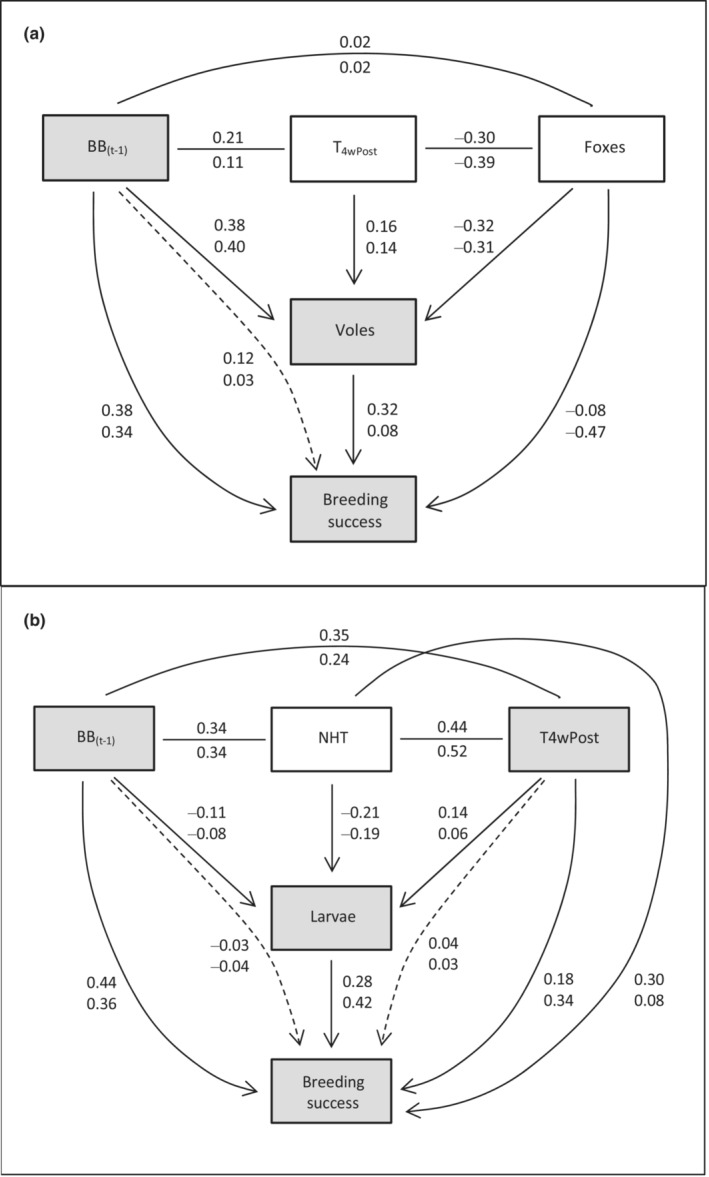
Path diagram showing direct and indirect effect sizes for variables in competing hypotheses. (a) The Predation Hypothesis (APH sub‐hypothesis: Voles) versus Chick Food Hypothesis (Plant Stress sub‐hypothesis: BB_(*t*−1)_). Indirect effect on breeding success shown with hatched line for BB_(*t*−1)_ via Voles. (b) The Chick Weather Hypothesis (T_4wPost_) and Chick Food Hypothesis (Larvae and BB_(*t*−1)_). Indirect effects on breeding success shown with hatched lines for BB_(*t*−1)_ and T_4wPost_ via Larvae. Effect sizes are shown with coefficients for capercaillie above black grouse. Detailed test statistics are shown in Appendix S6: Tables 1–3.

Path analyses helped to identify possible causal relationships among confounded variables from the competing Predation, Chick Food, and Chick Weather Hypotheses. First, in the Chick Food Hypothesis, breeding success was hypothesized to be influenced by the previous year's bilberry crop directly via the plant's nutritive value. Path analysis modeled the alternative possibility that previous year's bilberry crop might exert an indirect effect on breeding success via vole abundance, which buffered predation by foxes (APH). Overall, the direct effect of bilberry was stronger than the indirect one via voles (Figure 5a, Appendix S6: Figure 1 and Table 1). However, although the Plant Stress sub‐hypothesis was upheld, a causal relationship between bilberry and breeding success via vole abundance was hinted at.

Second, we looked for indirect effects of bilberry and temperature on breeding success via larvae. Although previous year's bilberry crop and present year's summer temperature both affected breeding success directly and positively, their indirect effects via larvae were negligible (Figure 5b, Appendix S6: Figure 2, Tables 2 and 3).

## 4 DISCUSSION

In grouse, previous studies of breeding success have examined only a few potentially causal factors simultaneously, and confounding effects among explanatory variables have not been considered. Here, we first discuss each hypothesis separately, inferring their explanatory strength based on whether their predictions were fulfilled or not—the hypothesis‐testing part (section 4.1). We also comment on the justification of some of the underlying assumptions. After that, we look at the combined set of explanatory variables, irrespective of the hypotheses about biological causation—the AICc model selection part—to see if this approach would shed new light on the relationships. It turned out that when we combined the best variables from each hypothesis, confounding was identified among key food and predation variables (section 4.2). Finally, we comment on the path analysis and discuss the inherent problem when inferring biological causation from correlative data (section 4.2).

### 4.1 Hypothesis testing

Our data, collected over 41 years, confirmed most of the predictions based on five current hypotheses about the determinants of breeding success in boreal forest grouse. Only the two sub‐hypotheses comprising the Demography Hypothesis were refuted. One—the Age‐dependence sub‐hypothesis (Lindström et al., 1997)—predicted low breeding success in years with a large proportion of first‐year hens. In our study area, all radio‐tagged capercaillie hens attempted breeding, and although the breeding output of first‐year birds was lower than among older hens (Storaas et al., 2000), the difference was small and apparently had little influence on annual variations in average breeding success. In the smaller black grouse, two large datasets have reported contrasting results; whereas Marjakangas and Törmälä (1997) did not find differences in reproductive outputs between yearling and older hens, Willebrand (1992) found much lower breeding success among yearlings. Clearly, our data were not consistent with the prediction of the Age‐dependence sub‐hypothesis.

Neither was the prediction that high density of hens should lead to lower reproductive output verified, possibly because densities of the two grouse species were below the carrying capacities of their habitats. Studies in Finland (Kauhala & Helle, 2002), NW Russia (Borchtchevski et al., 2003), and Scotland (Summers et al., 2010) all reported slightly higher breeding densities than in our study area, but no study of forest grouse has yet examined the relationship between bird density and carrying capacity.

The Hen Condition Hypothesis gained some support, especially in capercaillie, which experienced higher brood frequencies in warmer springs, confirming the findings of Wegge and Rolstad (2017). In both species, poor breeding with low brood frequencies occurred after winters with high winter NAO indices, which went along with mild and moist local weather. Possibly, by preventing snow burrowing, such weather may have affected thermoregulation and hen condition negatively, thereby causing less investment in breeding. Notably, however, none of the local winter weather variables explained any variation in breeding success: Hallett et al. (2004) and Stenseth and Mysterud (2005) discussed similar discrepancies between effects of regional and local weather, concluding that large‐scale weather indices may include biologically influential weather aspects not recorded by standard meteorological measurements.

A few studies have used the NAO index as explanatory variable in their analyses. In line with our study, Vergara et al. (2012) suspected red grouse in Scotland to be in better condition after winters with low winter NAO indices, as they found males (but not females) to have larger combs the following spring. Barnagaud et al. (2011) found a nonlinear n‐shaped relationship between winter NAO and the breeding success of black grouse in the French Alps. Finally, Kvasnes et al. (2014) found a positive correlation between NAO and the breeding success of willow ptarmigan in Norway, although this involved spring and summer NAO indices. Thus, interconnections among large‐scale weather indices, local weather measurements, and grouse performance remain unresolved.

The date of snow‐free ground—a proxy for the timing of new plant growth in spring—did not seem to influence breeding performance. We were surprised at this, expecting warm spring weather and access to bilberry and early sprouting bog cottongrass (*Eriophorum vaginatum*) (Odden et al., 2003; Pulliainen & Tunkkari, 1991) to increase chick production. The absence of detectable effects could be explained by NAO and time of snow melt being negatively correlated—positive NAO winters (warm and moist) often were followed by early springs (Appendix S2: Table 2). Also, temporal variation in bilberry fruiting and changing levels of chemical deterrents in early bilberry shoots (as in the Plant Stress sub‐hypothesis) may have affected the physical condition of breeding hens.

The Chick Weather Hypothesis received some support, but markedly less than Chick Food and Predation. As in other studies (Baines et al., 2016; Ludwig et al., 2010; Moss et al., 2001; Wegge & Rolstad, 2017), warm weather after hatching had a positive effect on breeding success. Although less influential than chick food, temperature after hatching was included in four of the six best AIC models of breeding success. In early life, chick foraging bouts are probably longer and more efficient during warm weather, when arthropod foods are more active and more readily detected. Contrary to some other studies (Baines et al., 2016; Coppes et al., 2021; Ellison & Magnani, 1984; Moss, 1986; Storch, 1994; Summers et al., 2004), rainy weather following hatching did not affect breeding success in our study. Nor was there any interaction between temperature and rain: the rain did not matter whether it was warm or cold. This was surprising and inexplicable, as our study area does not differ much from the weather regime in the aforementioned studies.

The Chick Food Hypothesis was well supported by our data. Firstly, the prediction that breeding success should be positively related to insect larvae was clearly confirmed. Their wide and irregular fluctuations explained breeding success well in both species. We had expected larval abundance primarily to affect survival of chicks and were therefore surprised that it also affected brood frequency. While whole broods may perish from starvation or predation (Ludwig et al., 2010; Marjakangas & Törmälä, 1997; Wegge & Kastdalen, 2008; Willebrand, 1992), brood frequency is mainly related to nest loss (Baines et al., 2016; Ludwig et al., 2010; Summers et al., 2009)—modified by renesting, as recorded in our study area (Storaas et al., 2000).

Secondly, the positive effect of bilberry fruiting the preceding year supported the Plant Stress sub‐hypothesis—the mast depression hypothesis of Selås (1997). His added prediction (Selås, Sonerud, et al., 2011), that cold summers in a bilberry masting year (and the year before) should accentuate the positive effects, also received some support via a weak effect on brood size. However, serious confounding between bilberry fruiting and vole abundance became apparent when the chick food and predation hypotheses were analyzed in concert (Table 6, see section 4.2). Finally, the positive confounding between bilberry and larvae (Table 6) makes sense on the basis that the two foods are likely to complement each other through their effects on chick diet quality.

Among the Predation sub‐hypotheses, the Alternative Prey hypothesis (APH) was well supported by a positive effect of vole abundance on breeding success (but see Table 6 and section 4.2 on confounding). APH has received much support in Scandinavia (section 1.1.4). Secondly, as predicted, the growth rate of foxes tracked vole abundance, and this may also have been the case for other mesopredators such as pine marten and stoat. Hence, the increase in fox numbers mediated by high vole abundances contributed to an increased predation pressure during the low phase of the vole cycle. This moderately negative effect was mainly through effects on brood frequency, indicating more predation on eggs in nests than on chicks in broods—consistent with quite high nest losses recorded in the study area (Storaas & Wegge, 1987).

The Delayed Raptor sub‐hypothesis (Tornberg et al., 2005) received some support in the hypothesis‐testing, and Grouse_(*t*−23)_—a surrogate for a delayed numerical response in goshawk—also figured in the three best AICc‐models with effect size stronger than Foxes and Voles. Although trends and possible cyclicity in breeding success are beyond the scope of this study, we observed that chick production fluctuated in a quasiperiodic pattern with intervals longer than expected solely from the 3–4 years vole cycle (Figure 1a). Such prolonged quasi‐cycles of 6–7 years are well documented from Finnish grouse populations (Lindström et al., 1995). Thus, a delayed effect of predation might be at work, resembling the classic Lotka‐Volterra predator–prey model (Odum, 1953). Tornberg et al. (2012) provided some evidence of a 2–3 years numerical lag in goshawk territory occupancy from Finland, and Selås and Kålås (2007) reported a weak 2 years lag from southern Norway. Apart from this, no other studies have provided convincing evidence for such a delayed numerical response in goshawk (e.g., Lindén, 1988).

Summing up, we evidenced several bottom‐up and top‐down factors influencing breeding success and noted that they fluctuated asynchronously. Furthermore, the two components of breeding success—brood frequency and brood size—were completely uncorrelated and affected differently by the explanatory variables. This resulted in overall breeding success fluctuating around low to moderate levels.

### 4.2 Confounding effects and biological causation

Notable confounding of key variables became apparent when we tried to select the best predictive models from the full set of explanatory variables via AICc. Foremost was the confounding of Voles by BB_(*t*−1)_ and Larvae (Table 6). Consistent with APH, voles alone provided the best predictive model for brood size, but confounding eliminated voles from the best predictive models for annual variation in brood frequency and total breeding success.

Naïve reliance on AICc while taking no account of biological insights can lead to misleading inferences about causal relationships (Anderson & Burnham, 2002; Cade, 2015). Hypothesis‐testing relies on biological insight but heedless reliance on it can also be misleading. For example, the result from model selection that brood frequency was affected more by larval abundance (after hatching) than by predation (largely on nests), makes little biological sense. Likewise, while the prediction of the Plant Stress sub‐hypothesis that previous year's bilberry production should be related to breeding success was clearly verified, path analysis showed that this was due in part to an indirect relationship via voles. It may have been that high bilberry fruit crops increased the overwinter survival of bank voles (Selås et al., 2021), which subsist mainly on bilberry shoots during winter (Hansson, 1983), thereby buffering predation on nests and chicks via APH.

There are other biological reasons for keeping an open mind about the relative importance of the Plant Stress sub‐hypothesis and APH. First, young chicks eat only a small proportion of bilberry leaves during their first critical weeks of life when their mortality is highest (Wegge & Kastdalen, 2007, 2008), so weakening the suggestion that variations in the nutritive value of bilberry affect their survival. Second, larvae feed on bilberry leaves and larval abundance did not vary with bilberry fruiting. Likewise, White (1984)—the originator of the Plant Stress Hypothesis—detected no positive responses in insectivorous herbivores feeding on stressed plants. Finally, Selås, Holand, and Ohlson (2011) reported no effect of fruiting on the digestibility or N‐content in shoots in bilberry plants.

### 4.3 Predation

The mean number of chicks reared per hen was 1.2 in capercaillie and 1.9 in black grouse. These had mean clutch sizes of 7.1 and 8.2, respectively, so that about 80% of potential recruits were lost during the 4‐month nesting and brood rearing period (Table 2b). Breeding success was determined partly by brood size but more by brood frequency, determined largely by red foxes and pine martens taking nests. Although incubating hens are well camouflaged and expose their eggs for only short periods during recesses, more than half of all nesting attempts fail (Wegge & Storaas, 1990). The generally low breeding success in our study area compared with other studies (Baines et al., 2016; Borchtchevski et al., 2003; Kurki et al., 1997; Marcström et al., 1988; Saniga, 2011) went along with rather dense populations of predators, especially red fox (Wegge et al., 2018).

Although we found predation to have a marked impact on breeding success, the importance of APH via Voles was lower than expected from previous studies in Scandinavia. Possible reasons are (1) the irruption of sarcoptic mange disease in red fox in the late 1980s, which depressed the abundance of foxes during a few years (Lindström et al., 1994; our study), and (2) low abundance of voles and absence of the regular 3–5‐year vole cycle during a long period in the early 2000s (Ims et al., 2008; our study).

In Fennoscandia, predation has long been considered an important cause of mortality in boreal forest grouse. Voiced largely by hunter organizations, this view has also been tested in field experiments via intensive predator control (Kauhala et al., 2000; Marcström et al., 1988) or provision of supplemental food (Finne et al., 2019; Lindström et al., 1987), all in the presence of vole cycles. In all four studies, breeding success—but not adult numbers—increased with treatments. Notably, these positive effects correlated with vole abundance—but only when predators were present: when main predators were scarce or absent, breeding success did not decline during the low phase of the vole cycle. Besides providing clear support for the APH mechanism, these studies document the strong effect of mammalian predation on breeding success in forest grouse. Its impact may well be stronger in more northern parts of Fennoscandian boreal forests because vole cycles are more pronounced there, showing larger amplitudes than at lower latitudes where prey are more diverse and abundant (Korpimȁki et al., 2005).

It should be noted that, in the present study, we have inferred possible causes of annual variations in breeding success from correlations with year‐to‐year variations in explanatory variables. This implies that variables with little yearly variation have less explanatory power than variables with large annual amplitudes. Predictions from both the Red Fox and the Delayed Raptor Hypothesis were tested with variables (Foxes and Grouse_(*t*−23)_) that varied relatively little over the years of the study, thereby reducing their statistical power to detect possible effects (see Figure 2c and d). Thus, although the role of predation in explaining *annual variation* in breeding success appeared rather modest, predation may still be the overall most important factor reducing nesting success and chick survival, as previously documented in our study area (Wegge & Kastdalen, 2007; Wegge & Storaas, 1990).

### 4.4 Breeding success and population regulation

We detected no density‐dependent effect on breeding success. The two grouse populations may have been regulated by density‐dependent losses of adults or juveniles rather than of eggs and chicks. If so, this is in line with the general conclusion reached by Sæther et al. (2016) from the population dynamics of 13 bird populations, supporting the population regulation hypothesis launched in the 1960s by David Lack (1966). Another generalization is that, although environmental stochasticity—mainly in food and weather—impacts all vital rates, it most strongly affects temporal variation in breeding success and recruitment (White, 2008). Hence, variation in breeding success should be determined primarily by environmental stochasticity rather than density dependence.

In populations where immigration and emigration cancel each other, variations in adult population size depend on adult mortality plus juvenile recruitment, the latter varying with breeding success and overwinter survival of fledged chicks (Moss & Watson, 2001). As a general rule, avian breeding success varies independently of density. However, exceptions have been reported, even within the same species: In song sparrows (*Melospiza melodia*), for example, Arcese et al. (1992) showed that density‐dependent reproductive success and density‐dependent recruitment of juveniles each operated to regulate population size in a sequential manner. In a different population of the same species, Chase et al. (2005) found that adult density was related to rainfall‐associated, density‐independent variations in reproductive success, plus density‐dependent losses of adults in the previous year. In our study area—despite long‐term increase in density‐independent breeding success—adult numbers of the two grouse species have changed very little (Wegge & Rolstad, 2011). This suggests that losses during juvenile recruitment and among adults may have increased in a density‐dependent fashion. However, since this scenario has not yet been examined statistically, we present no direct evidence of this.

## 5 CONCLUSION

Barring hypotheses about demographic characteristics, all the other four hypotheses—Hen Condition, Chick Weather, Chick Food, and Predation—were supported. Some predictions were not verified, most surprisingly the expected negative effect of precipitation in the Chick Weather hypothesis. Model selection across hypotheses indicated that bottom‐up factors (bilberry and larvae) may be more important than weather and top‐down factors—i.e., numerical and functional effects of predation—in driving annual fluctuations in grouse breeding success. Notably, the strong effect of insect larvae as chick food was consistently present in all analyses. Confounding among key variables, including bilberry crop the previous year, vole abundance, and larval abundance, complicated the interpretation of causal relationships, but path analysis suggested that bilberry may have acted in part through an effect on vole abundance, which buffered predation. The lack of any influence of density or age composition of breeding hens indicated that density‐dependent factors played little or no role in breeding output.

Importantly, our findings are based on correlations and not experimental evidence. To unravel causal relationships, field experiments and well‐designed comparative studies are needed, especially directed at the functional roles of bilberry fruiting vis‐a‐vis voles and larvae chick food. Another unresolved issue relates to the rather strong and consistent effect size of the winter NAO index. Since we did not find any direct effects of local winter weather variables, the ecological mechanism explaining this correlation remains unknown. Finally, a possible delayed predation effect from goshawks is at best tentative, since we did not have reliable data on goshawk numbers.

## AUTHOR CONTRIBUTIONS

**Per Wegge:** Conceptualization (lead); data curation (lead); formal analysis (equal); funding acquisition (lead); investigation (lead); methodology (lead); project administration (lead); resources (lead); software (supporting); supervision (lead); validation (equal); visualization (supporting); writing – original draft (lead); writing – review and editing (equal). **Robert Moss:** Formal analysis (lead); methodology (equal); software (lead); writing – review and editing (supporting). **Jorund Rolstad:** Formal analysis (supporting); funding acquisition (supporting); investigation (supporting); methodology (supporting); project administration (supporting); resources (supporting); validation (supporting); visualization (lead); writing – original draft (supporting); writing – review and editing (supporting).

## Supporting information


Appendix S1

## Data Availability

Relevant data files are archived in the Dryad Digital Repository: https://doi.org/10.5061/dryad.931zcrjpb.
