# Supplementary material for: Annual variation in breeding success in boreal forest grouse: Four decades of monitoring reveals bottom‐up drivers to be more important than predation
Source: Ecol Evol. 2022 Oct 9;12(10):e9327. doi: 10.1002/ece3.9327 (PMC9548575; doi:10.1002/ece3.9327)
Supplement: Supplementary file 1 — Appendix S1 [file ECE3-12-e9327-s001.pdf]

Supporting Information to:

Annual variation in breeding success in boreal forest grouse: four decades of monitoring reveals bottom-up drivers to be more important than predation

Per Wegge, Robert Moss & Jørund Rolstad

Ecology and Evolution, 2022

Appendix A: Study area and data collection. .... p. 2

Appendix B: Explanatory variables and correlation matrix. .... p. 7

Appendix C: Breeding success, brood frequency and brood size
- relationships within and between species. .... p. 10

Appendix D: Winter and summer weather relationships. .... p. 12

Appendix E: Species-specific effects on breeding success of explanatory variables.
Hypotheses tested separately for each species. .... p. 14

Appendix F: Path analyses showing direct and indirect effect sizes of confounded
explanatory variables in the Chick Food and Predation Hypotheses. .... p. 15

Appendix A: Study area and data collection

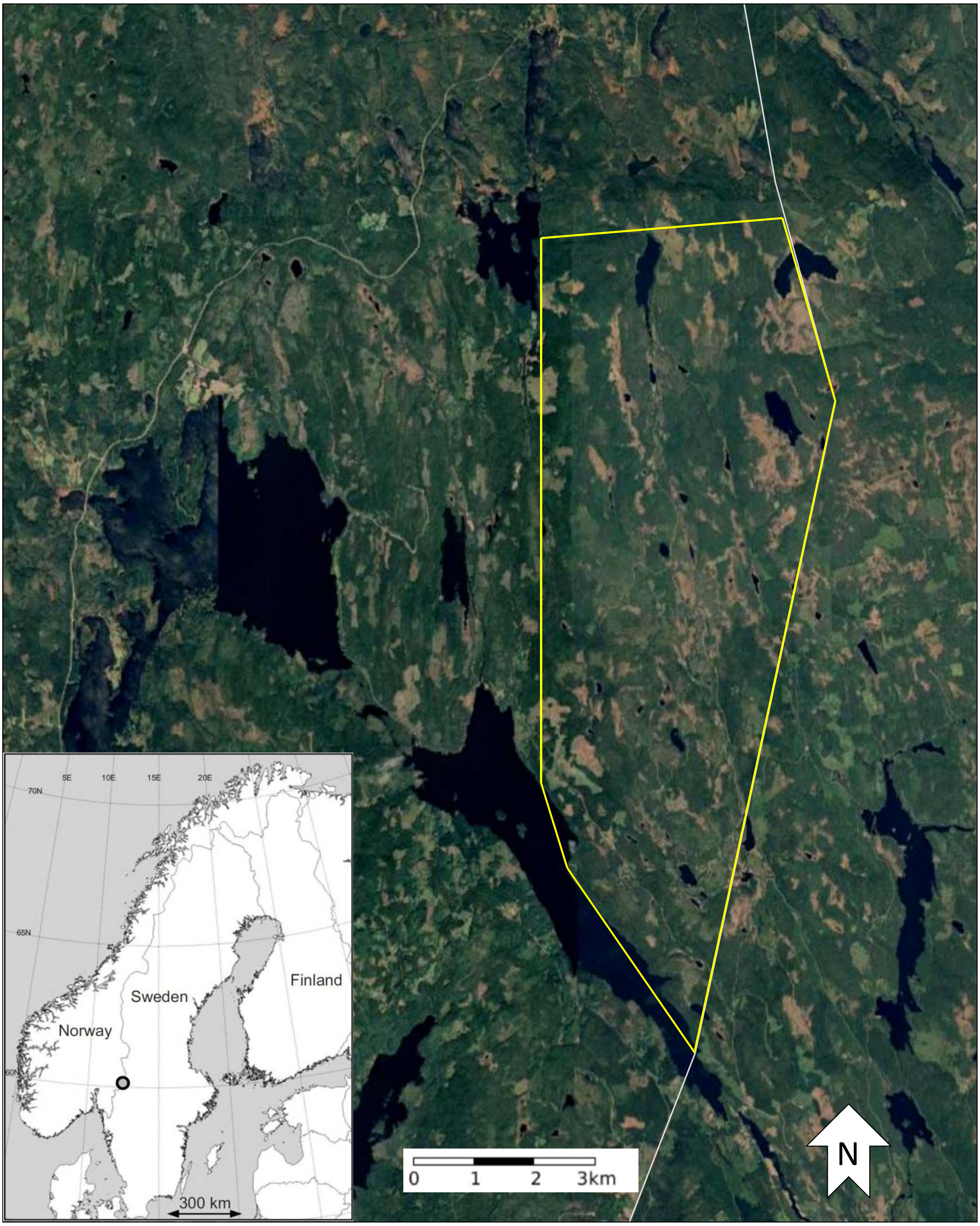

FIGURE A1. The 45 km^2 study area at Varald State Forest (yellow line), on the border to Sweden (white line). (60°10' N, 12°30' E).

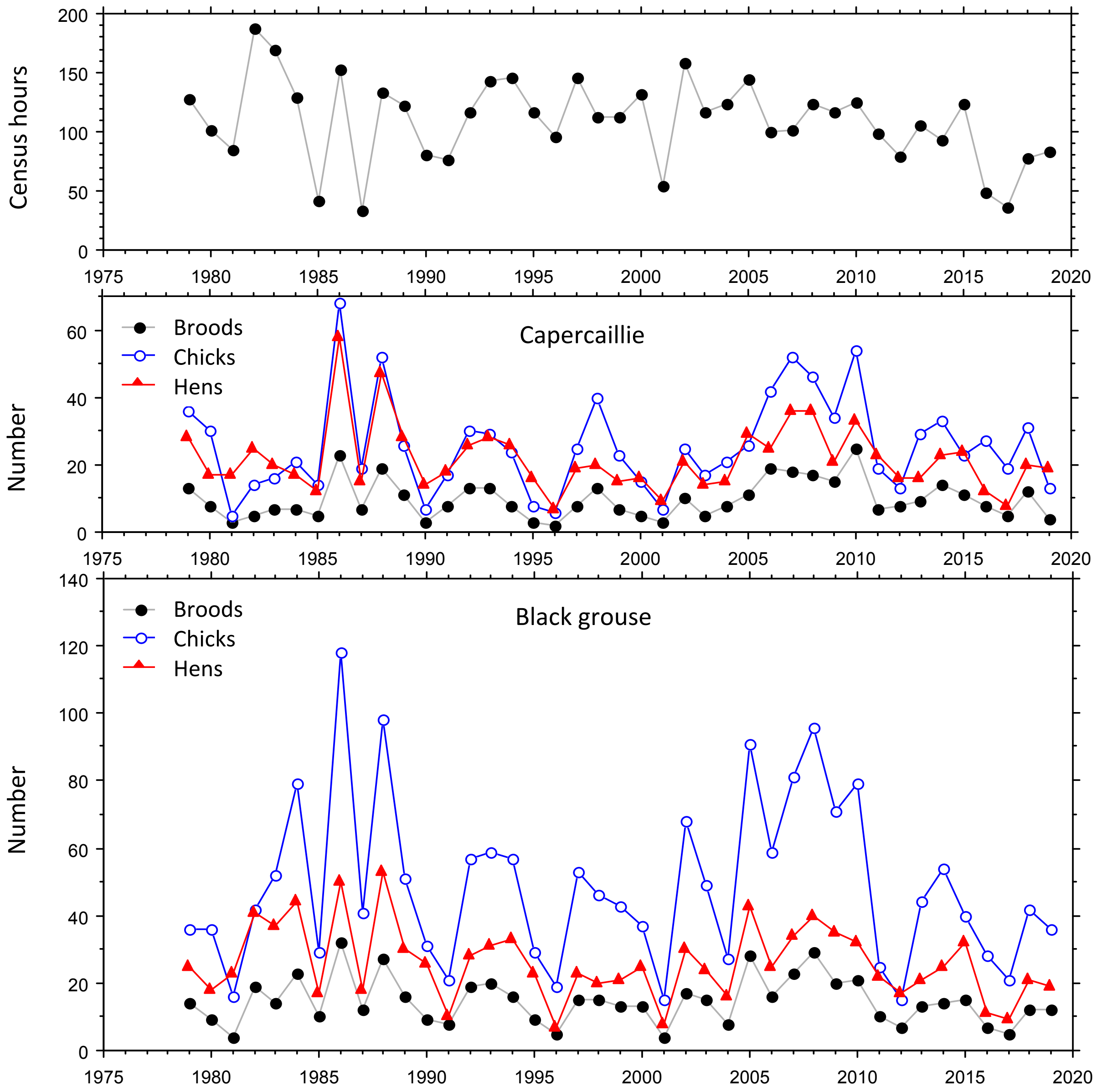

FIGURE A2. Census effort (census hours) and sample size of hens, chicks and broods each year.

Appendix A1, Sampling of birds and brood frequency and brood size: Each year, man-dog teams searched for birds within twenty-two 1.8–2.3 km^2 sized census blocks distributed across the 45-km^2 study area. Man-dog teams did not sample along fixed, straight transects, but meandered through the block, searching for birds in habitats suspecting to find birds. Most blocks were sampled once each year with search time varying between 3.8 and 6.3 hours per block. Due to logging, some blocks were discontinued and replaced by similar habitat-blocks nearby. Most blocks abutted each other. To minimize double counting of birds being flushed into neighbouring blocks, each observer marked on a map the route walked and the location, time and flight direction of the flushed birds. Sampling time and number of recorded birds varied between years, averaging 109 hours and 76 adult birds per year. Number of broods per year varied between 3 and 25, averaging 9.8 (capercaillie) and 4 and 32, averaging 14.6 (black grouse). Flushed birds were classified to species, sex (of adults), and number of chicks in broods. In capercaillie, we also recorded the sex of clearly seen chicks (sexual difference in plumage colour); this was not attempted in black grouse due to small sexual difference in August chick plumage in this species. Based on the proportions of sex-classified capercaillie chicks in August (177 males and 309 females), and a more equal sex-ratio of broods in good production years, the frequency of female chicks in years of small sample size was estimated by model fitting from known broods: *freq female* = 0.85 − 0.19 *ln* (brood size), averaging 66:34 in favour of hens. For black grouse, we used a ratio of 55:45 based on Hörnfeldt et al. (2001).

Counts of hens in August were used as a surrogate for breeding density in spring because intervening summer mortality of hens is negligible (Wegge & Rolstad, 2011). Most juvenile hens attempted to breed (Storaas et al., 2000). Although juveniles have been inferred to suffer higher over-winter mortality than older hens (Hannon & Martin, 2006; Pekkola et al., 2014), we used the proportion of female chicks amongst all hens seen in August as a surrogate for the proportion of young hens in spring the following year. We used number of observed birds in different categories per 10 hours of sampling time as an index of density. Among all birds recorded (6739), unclassified individuals comprised 17 percent. These were ignored in the analyses since their proportion varied little among years. Date of peak of mating was estimated from direct observations at leks, supplemented by monitoring 4–6 leks with remote cameras since 2015.

Breeding success is the ratio of number of chicks and number of hens counted during sampling with pointing dogs in August. It consists of two components: the proportion of hens accompanied by at least one chick (brood frequency) and number of chicks in the broods (brood size). Reflecting mainly survival of nests and chicks, respectively, their estimates − in this respect − will be biased due to factors like detection probability of broods and solitary hens, non-breeding yearlings, renesting, nest desertion, and loss of whole broods. However, due to small and mostly opposite effects on the frequency and size of broods, they largely nullify each other. An exception is loss of whole broods, which can be quite high (Willebrand, 1992; Marjakangas & Törmälä, 1997 (black grouse), and Storch, 1994; Wegge & Kastdalen 2007 (capercaillie)). Therefore, nest survival will − to a greater or lesser degree − be higher than the estimated brood frequency, and chick survival will be lower than indicated by the estimated brood size. There were no correlations between annual census hours and breeding success, neither for the two species nor for the two components of brood frequency and size (r = (-0.19) − (-0.07), p = 0.22–0.66).

To enable a comparison of the relative contribution of brood frequency and brood size to the overall breeding success, we used a clutch size of 7.1 eggs in capercaillie and 8.2 eggs in black grouse (Wegge, 1979, and unpublished from Varald State Forest 1979–1996).

Appendix A2, Weather data: Local weather statistics were downloaded from Kongsvinger meteorological station (eKlima at http://sharki.oslo.dnmi.no/) 25 km from the study area. The station-based winter NAO index was downloaded from NOAA's Climate Prediction Center (www.cpc.ncep.noaa.gov/

products/precip/CWlink/ pna/nao.shtml). NHT (HadCRUT4nh) was down-loaded from the Climatic Research Unit of UEA-CRU (crudata.uea.ac.uk/ cru/data/temperature/).

Appendix A3, Predation data: Voles were sampled in autumn (late August – early September) using snap traps, baited with raw potato, along 6 fixed transects in two types of habitats: bilberry-dominated, old spruce forest, which is the prime habitat of bank voles (Gorini et al., 2011), and grass-dominated successions 3 to 8 years after clearcutting, the prime habitat of field voles (Hansson, 1983). In each habitat, we trapped voles along transects at 2–3 different sites each year. Logging and regrowth of clear cuts necessitated relocation of some transects during the study. At each transect, 50–100 traps were set 5 m apart in late afternoon for 2 nights, amounting to 350-600 trap-nights per year (Wegge & Rolstad, 2018). Yearly abundance indices were calculated for each habitat type and expressed as number of voles captured per 100 trap-nights. Bank voles dominated in both habitats with a mean annual trapping index of 6.4/100 trap nights versus 2.1 for field voles in early, grass-dominated successions, and 8.3 and 0.03 in old bilberry forest, respectively.

Red fox harvest statistics were converted to winter track counts based on mean ratios of the two indices available in the same years. The track index was expressed as numbers of track crossings per 24 hours per 10 km transect length. Foxes often travel along roads and trails before crossing. We therefore omitted “new” tracks that crossed back within 200 m of the first encounter.

Appendix A4, Chick food data: Larvae were sampled in late May and June by sweep netting at fixed stations in bilberry-rich sites of old spruce-dominated stand. (Owing to logging disturbance, number and location of stations had to be changed sometimes). We used a net measuring 36 cm in diameter on a 2 m long pole. Ten-fifteen sweeps were made at each site in the shrub layer while walking slowly forward, sweeping a distance of c. 1.5 m per sweep. Lepidopteran and Hymenopteran larvae were counted and grouped into three size classes: small (<5 mm in length), medium (5–12 mm) and large (>12 mm). The diameter of some larvae deviated markedly from the rest; in such cases thick ones were upgraded to a larger size class, and very slim ones downgraded to a lower class. The larval index was expressed as numbers of large and medium sized larvae per 10 sweeps. Each station was sampled at 4–7 days intervals over a period of 3–4 weeks (Wegge et al., 2010). The abundance of larvae available to chicks each year was estimated as follows. First, we estimated hatching dates from the peak mating dates. We then interpolated the abundance indices of large and middle-sized larvae to the period of 8 to 12 days post-hatch. Capercaillie chicks hatch a few days earlier, but subsist on larvae for a longer period than black grouse (Wegge & Kastdalen, 2008). Hence, the estimated abundance indices correspond closely to the critical periods of larvae food for both species. The data set of larvae covered 28 years, irregularly until 2002, thereafter annually.

Fruits of bilberry (berries) were counted during the last 17 years (2003-2019) within three 0.5 m^2 circular plots randomly distributed within a radius of 10 m at 6–8 fixed stations in bilberry-rich, old spruce forest in August. As for larvae and voles, a few stations had to be relocated due to clear cutting. At each plot, we visually estimated the horizontal coverage of ramets and counted the number of berries. After correcting for ramet coverage, berry abundance was indexed as numbers per m^2 of bilberry plants. We also had access to bilberry fruit indices in the study area for the whole period of 41 years, based on newspaper records (Selås et al., 2021). The newspaper indices for the same period were correlated with our own field measurements during the same time period ($r_s = 0.50$, $p = 0.047$, $n = 17$). Also, parallel analyses for the final 17 years using either our field measurements or the newspaper index gave very similar results. We therefore use the latter in our statistical analyses.

References

Gorini, L., Linnell, J.D., Boitani, L., Hauptmann, U., Odden, M., Wegge, P., & Nilsen E.B. (2011). *Integrative Zoology, 6,* 299-310.

Hannon, S.J., & Martin, K. (2006). Ecology of juvenile grouse during the transition to adulthood. *Journal of Zoology, 269,* 422-433. https://doi.org/ 10.1111/j.1469-7998.2006.00159.x.

Hansson, L. (1983). Competition between rodents in successional stages of taiga forests: *Microtus agrestis* vs. *Clethrionomys glareolus*. *Oikos, 40,* 258-266.

Hörnfeldt , B., Hipkiss, T., & Eklund, U. (2001). Juvenile sex ratio in relation to breeding success in Capercaillie *Tetrao urogallus* and Black Grouse *T. tetrix. Ibis, 143,* 627-631.

Marjakangas, A. & Törmäla, L. (1997). Female age and breeding performance in a cyclic population of black grouse *Tetrao tetrix*. *Wildlife Biology, 3(3/4),* 195-203. https://doi.org/10.2981/wlb.1997.024

Pekkola, M., Alatalo, R., Pöysä, H., & Siitari, H. 2014. Seasonal survival of young and adult black grouse females in boreal forests. *European Journal of Wildlife Research, 60,* 477-488.

Selås, V., Framstad, E., Rolstad, J., Sonerud, G.A., Spidsø, T.K., & Wegge, P. (2021). Bilberry seed production explains spatiotemporal synchronicity in bank vole population fluctuations. *Ecological Research,* https://doi.org/10.1111/1440-1703.12204.

Storaas, T., Wegge, P., & Kastdalen, L. (2000). Weight-related renesting in capercaillie. *Wildlife Biology, 6,* 299-303.

Storch, I. (1994). Habitat and survival of capercaillie *Tetrao urogallus* nests and broods in the Bavarian alps. *Biological Conservation, 70*, 237-243.

Wegge, P. (1979: Status of capercaillie and black grouse in Norway. In: Lovel, T. (Ed.); Proceedings *of 1. International Symposium on Grouse, WPA, Suffolk, 1978,* 17–26.

Wegge, P., & Kastdalen, L. (2007). Patterns and causes of natural mortality of capercaillie Tetrao urogallus chicks in fragmented boreal forest. *Annales Zoologici Fennici, 44,* 141-151.

Wegge, P., & Kastdalen, L. (2008). Habitat and diet of young grouse broods: resource partitioning between Capercaillie (*Tetrao urogallus*) and Black grouse (*Tetrao tetrix*) in boreal forests. *Journal of Ornithology, 149,* 237-244.

Wegge, P., & Rolstad, J. (2011). Clearcutting forestry and Eurasian boreal forest grouse: long-term monitoring of sympatric capercaillie *Tetrao urogallus* and black grouse *T. tetrix* reveals unexpected effects on their population performances. *Forest Ecology and Management, 261,* 1520-1529.

Wegge, P., & Rolstad, J. (2018). Cyclic small rodents in boreal forests and the effects of even-aged forest management: patterns and predictions from a long-term study in southeastern Norway. *Forest Ecology and Management, 422,* 79-86.

Wegge, P., Vesterås, T., & Rolstad, J. (2010). Is the timing of breeding and subsequent hatching in boreal forest grouse adapted to insect food for the chicks? *Annales Zoologici Fennici, 47,* 251-260.

Willebrand, T. (1992). Breeding and age in female black grouse *Tetrao tetrix. Ornis Scandinavica, 23,* 29-32.

Appendix B: Explanatory variables and correlation matrix.

TABLE B1. Complete list of variables used in the initial screening for analysis. Response, detrending (NHT) and the 13 explanatory variables selected for the main analyses, are marked with a black dot.

Main hypotheses	Sub-hypotheses		Variables	Description
		•	Breeding success	Number og chicks per hen
		•	Brood frequency	Proportion of hens with brood
		•	Brood size	Number of chicks per hen with brood
		•	NHT	Average yearly temperature (°C) in the Northern Hemisphere, used as detrending variable
**Hen Condition**			Tav_{winter}	Average daily mean temperature (°C) during winter months (DJFM)
**Hen Condition**			$Prec_{winter}$	Average daily precipitation (mm) during winter months (DJFM)
**Hen Condition**			SnowDepth	Average daily snow depth (cm) during winter (JFM)
**Hen Condition**			SnowMax	Maximum snow depth (cm) during winter (DJFM)
**Hen Condition**			SnowDays	Number of days with snow depth ≥ 30 cm during winter (DJFM)
**Hen Condition**			$PrecRain_{winter}$	Average daily precipitation (mm) coming as rain during winter months (DJFM)
**Hen Condition**			$PrecRainDays_{winter}$	Number of days with rain during winter (DJFM)
**Hen Condition**		•	NAO_{w}	Average North Atlantic Oscillation Index during winter months (DJFM). The difference of atmospheric pressure at sea level (SLP) between the Icelandic Low and the Azores High
**Hen Condition**			NAO_{spring}	Average North Atlantic Oscillation Index during spring months (MAM)
**Hen Condition**		•	T_{8wPre}	Average daily minimum temperature (°C) during 8 weeks prior to date of hatching
**Hen Condition**			T_{4wPre}	Average daily minimum temperature (°C) during 4 weeks prior to date of hatching
**Hen Condition**			$Temp_{April}$	Average daily minimum temperature (°C) during April
**Hen Condition**			$Temp_{May}$	Average daily minimum temperature (°C) during May
**Hen Condition**			$Temp_{April-May}$	Average daily minimum temperature (°C) during April-May
**Hen Condition**			$Precip_{April-May}$	Average daily precipitation (mm) during April-May
**Hen Condition**			FrostFree	Date of frost-free nights in late winter, calculated as the number of days after April 1 when 7 days running minimum temperature > 0 °C
**Hen Condition**		•	SnowFree	Date of snow-free ground, calculated as the number of days after April 1 when snow cover is < 20%
**Chick Weather**			T_{2wPost}	Average daily minimum temperature (°C) during 2 weeks after date of hatching
**Chick Weather**		•	T_{4wPost}	Average daily minimum temperature (°C) during 4 weeks after date of hatching
**Chick Weather**			$Temp_{June}$	Average daily minimum temperature (°C) during June
**Chick Weather**			$Temp_{JJA}$	Average daily mean temperature (°C) during current summer months (JJA)
**Chick Weather**			P_{2wPost}	Average daily precipitation (mm) during 2 weeks after date of hatching
**Chick Weather**			$PrecFreq_{2wPost}$	Frequency of days with rain (≥ 0.1 mm) during 2 weeks after date of hatching
**Chick Weather**		•	P_{4wPost}	Average daily precipitation (mm) during 4 weeks after date of hatching
**Chick Weather**			$PrecFreq_{4wPost}$	Frequency of days with rain (≥ 0.1 mm) during 4 weeks after date of hatching
**Chick Weather**			$Prec_{JJA}$	Average daily precipitation (mm) during current summer months (JJA)
**Chick Weather**			NAO_{summer}	Average North Atlantic Oscillation Index during summer months (JJA)
**Chick Food**	Food Quantity	•	Larvae	Abundance of larvae: Number of large and medium sized Lepidoptera and Hymenoptera larvae per 10 net-sweeps in bilberry-rich sites of old spruce forest
**Chick Food**	Food Quality		BB-Field$_{(t)}$	Field records of bilberry fruit abundance, indexed as numberof berries per 1 m2 of bilberry plants counted at 6-8 fixed stations in bilberry-rich, old spruce forest in August
**Chick Food**	Food Quality		BB-Field$_{(t-1)}$	Field records of bilberry fruit abundance previous summer
**Chick Food**	Food Quality		$BB_{(t)}$	Index of bilberry fruit abundance current year based on newspaper records. See Appendix A4 for more details
**Chick Food**	Food Quality	•	$BB_{(t-1)}$	Index of bilberry fruit abundance previous year based on newspaper records. See Appendix A4 for more details
**Chick Food**	Food Quality		$T_{JJA(t-1)}$	Average daily mean temperature (°C) during previous summer (JJA)
**Chick Food**	Food Quality		$T_{JJA(t-2)}$	Average daily mean temperature (°C) during the second past summer (JJA)
**Chick Food**	Food Quality	•	$T_{JJA(t-12)}$	Average daily mean temperature (°C) during the past two summers (JJA)

**Predation**	Alternative Prey	•	Voles	Number of voles per 100 trap-nights in August-September, averaged transects in old bilberry spruce forest and clearcuts
**Predation**	Alternative Prey		BVOF	Number of bank voles (Myodes glareolus) per 100 trap-nights in August-September, averaged transects in old bilberry spruce forest and clearcuts
**Predation**	Alternative Prey	•	Fox_{λ}	Relative change of fox abundance from previous winter till next winter. See Methods (Appendix 3) for details
**Predation**	Red Fox	•	Foxes	Red fox abundance index, based on snow tracks and harvest statistics. See Methods (Appendix 3 for details
**Predation**	Delayed Raptor		$Grouse_{(t-1)}$	Total density of grouse (numbers per 10 h census) previous year, used as a surrogate for the abundance of breeding godhawks
**Predation**	Delayed Raptor		$Grouse_{(t-2)}$	Total density of grouse (numbers per 10 h census) the second past year, used as a surrogate for the abundance of breeding godhawks
**Predation**	Delayed Raptor		$Grouse_{(t-3)}$	Total density of grouse (numbers per 10 h census) the third past year, used as a surrogate for the abundance of breeding godhawks
**Predation**	Delayed Raptor	•	$Grouse_{(t-23)}$	Total density of grouse (numbers per 10 h census) averaged over the second and third past year, used as a surrogate for the abundance of breeding godhawks
**Demography**	Age-dependence	•	YoungHen	Proportion of young hens in the breeding population, estimated as the proportion of female chicks of all females in previous years August census
**Demography**	Density-dependence	•	DensHen	Density (number/10 h census) of adult hens in August

TABLE B2. Cross-correlation matrix of the 13 explanatory variables used in the hypothesis tests, including NHT that was used for detrending. Pearson correlation coefficients on raw data at lower left and two-tailed *p*-values at upper right.

Main Hypothesis			Hen condition			Chick weather		Predation			Chick Food			Demography	
Sub-hypothesis								*APH*	*Red Fox*	*DRH*	*Food Quantity*	*Plant Stress*		*Age-dependence*	*Density-dependence*
Variables	(years)	NHT Detrending	NAO_w	T_{8wPre}	SnowFree	T_{4wPost}	P_{4wPost}	Voles	Foxes	$Grouse_{(t-23)}$	Larvae	$BB_{(t-1)}$	$T\_JJA_{(t-12)}$	YoungHen	DensHen
NHT	(41)	1	0.983	0.110	0.172	0.001	0.325	0.178	0.041	0.864	0.214	0.345	0.000	0.010	0.564
NAO_w	(41)	-0.003	1	0.891	0.046	0.192	0.179	0.546	0.549	0.718	0.665	0.582	0.624	0.729	0.804
T_{8wPre}	(41)	0.254	0.022	1	0.989	0.029	0.425	0.499	0.590	0.277	0.422	0.035	0.084	0.586	0.624
Snow-free	(41)	-0.218	**-0.313**	-0.002	1	0.514	0.638	0.723	0.144	0.176	0.935	0.977	0.133	0.134	0.065
T_{4wPost}	(41)	**0.492**	-0.209	**0.341**	0.106	1	0.409	0.050	0.009	0.854	0.532	0.307	0.151	0.004	0.143
P_{4wPost}	(41)	-0.158	-0.215	-0.129	0.076	-0.133	1	0.489	0.635	0.963	0.843	0.791	0.888	0.790	0.558
Voles	(41)	0.215	-0.098	0.109	0.057	**0.308**	0.112	1	0.177	0.705	0.205	0.001	0.790	0.203	0.192
Foxes	(41)	**-0.320**	0.097	0.087	0.233	**-0.401**	0.077	-0.215	1	0.055	0.148	0.645	0.348	0.047	0.065
$Grouse_{(t-23)}$	(38)	-0.029	0.061	-0.182	-0.225	0.031	-0.008	0.064	-0.313	1	0.02	0.192	0.549	0.987	0.066
Larvae	(28)	-0.243	-0.086	-0.159	-0.016	-0.124	0.040	0.248	-0.282	**0.444**	1	0.541	0.945	0.976	0.004
$BB_{(t-1)}$	(41)	0.152	-0.089	**0.329**	0.005	0.164	0.043	**0.481**	-0.075	-0.217	-0.122	1	0.282	0.182	0.235
$T\_JJA_{(t-12)}$	(41)	**0.534**	-0.079	0.273	-0.239	0.229	0.023	-0.043	-0.151	-0.101	-0.014	0.173	1	0.083	0.650
YoungHen	(40)	**0.402**	0.057	0.089	0.241	**0.445**	-0.044	0.206	**-0.316**	0.003	0.006	0.216	0.278	1	0.020
DensHen	(41)	-0.093	0.040	-0.079	0.291	0.233	-0.095	0.209	-0.291	0.301	**0.519**	-0.190	0.073	**0.365**	1

Appendix C: Breeding success, brood frequency and brood size relationships within and between species.

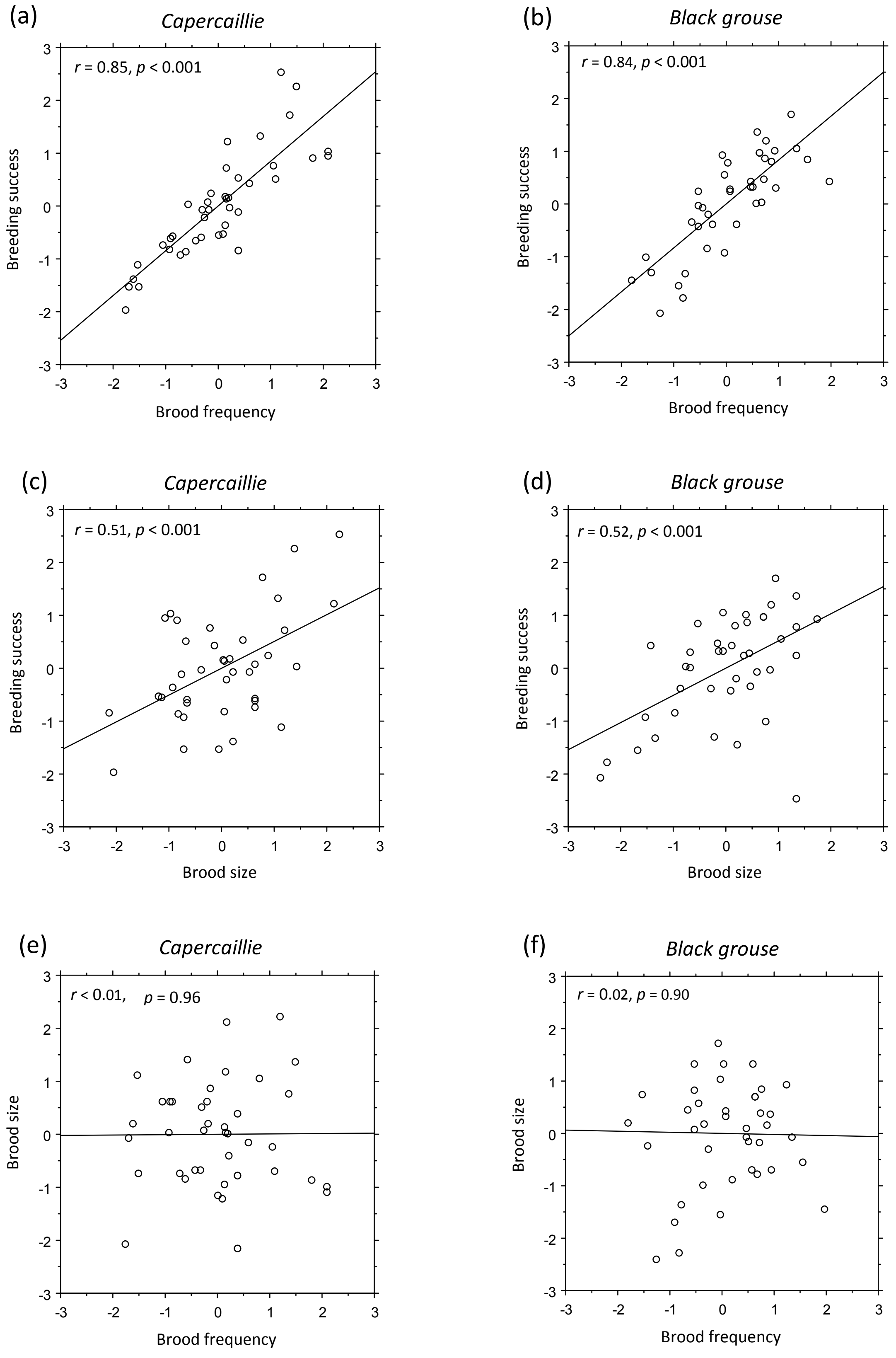

FIGURE C1. Correlations between breeding success, brood frequency and brood size within (a-f) and between (g-i) species. Based on standardised values (Z-scores). Pearson correlation coefficients with two-tailed *p*-values.

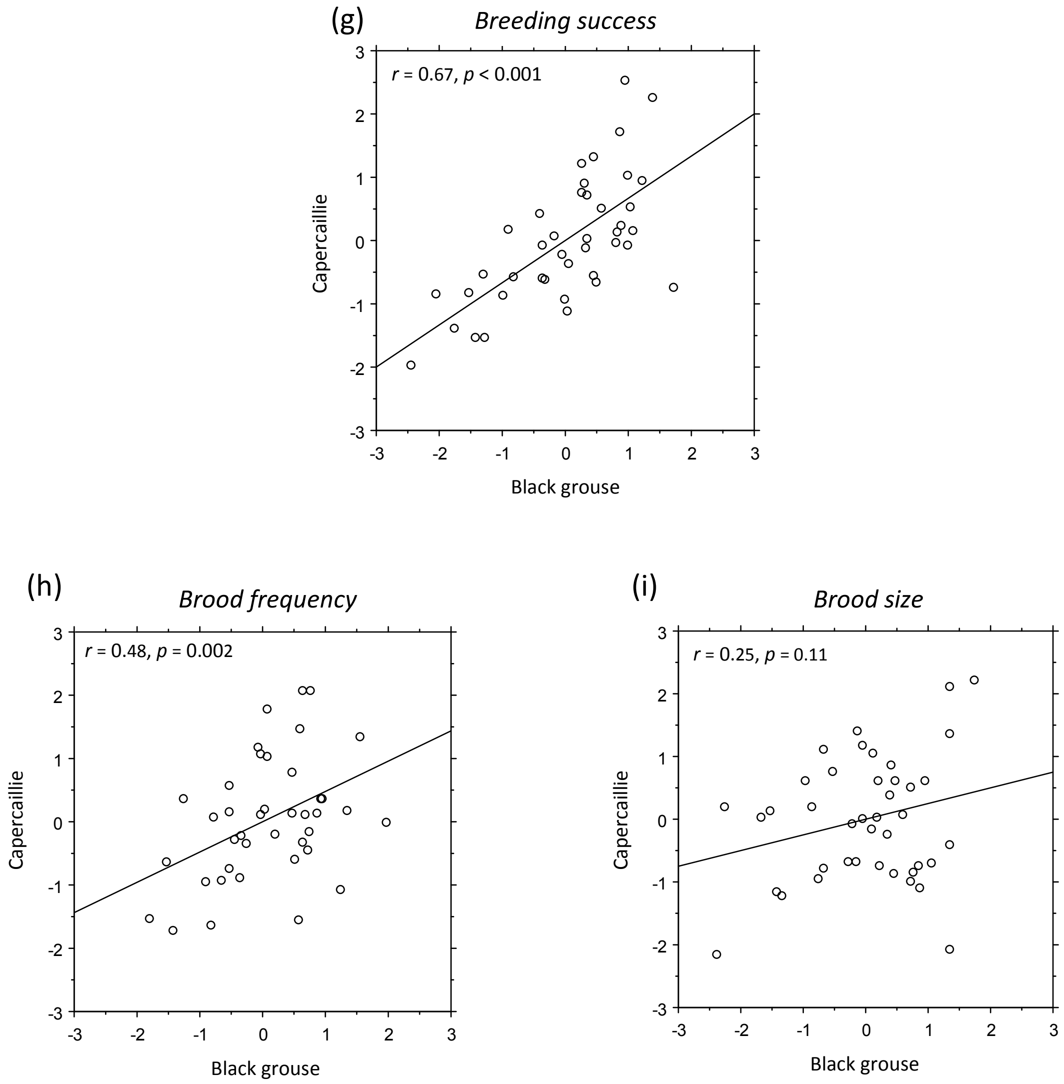

Figure C1 continued.

Appendix D: Winter and summer weather relationships.

TABLE D1. (a) Strength of associations (standardised partial regression slopes *β*, with *p*-values, two-tailed) between aspects of breeding success and winter weather. Models include data from both capercaillie and black grouse (n = 82). (b) Pearson correlation coefficients (*r*, with *p*-values, two-tailed) between NAO_w and local winter weather variables.

	NAO_w [a)]		Tav_{winter} [b)]		$Prec_{winter}$ [c)]		SnowDepth [d)]	
**(a)**	*β*	*p*	*β*	*p*	*β*	*p*	*β*	*p*
Breeding success	**-0.35**	*0.002*	<0.01	*0.973*	<0.01	*0.949*	-0.10	*0.362*
Brood frequency	**-0.32**	*0.003*	0.04	*0.734*	0.05	*0.638*	-0.10	*0.357*
Brood size	-0.15	*0.176*	-0.10	*0.376*	-0.12	*0.284*	0.02	*0.869*
Breeding success $_{partial}$ [e)]	**-0.34**	*0.001*	-0.15	*0.196*	-0.05	*0.658*	-0.02	*0.883*
Brood frequency $_{partial}$	**-0.32**	*0.002*	-0.08	*0.483*	<0.01	*0.962*	-0.03	*0.798*
Brood size $_{partial}$	-0.15	*0.177*	-0.17	*0.161*	-0.14	*0.206*	0.05	*0.647*
**(b)**	*r*	*p*	*r*	*p*	*r*	*p*	*r*	*p*
Tav_{winter}	**0.71**	*<0.001*		—		—		—
$Prec_{winter}$	**0.63**	*<0.001*	**0.83**	*<0.001*		—		—
Snow Depth	**-0.41**	*0.008*	**-0.81**	*<0.001*	**-0.78**	*<0.001*		—

a) The winter index of the North Atlantic Oscillation (DJFM).
b) Mean daily temperature averaged during winter months (DJFM).
c) Daily amount of precipitation averaged during winter months (DJFM).
d) Daily snow depth averaged during winter (DJF).
e) Partialling out Northern Hemisphere Temperature (NHT).

TABLE D2. ANOVA table of weather variables in the Chick Weather Hypothesis showing no interaction between temperature and rain during the four weeks period after hatching. (Two-tailed *p*-values, significant values boldfaced).

Effect	Breeding success		Brood frequency		Brood size	
	$F_{1, 72}$	*p*	$F_{1, 72}$	*p*	$F_{1, 72}$	*p*
Species	0.23	0.634	0.02	0.892	0.37	0.547
NHT	2.16	0.146	2.64	0.108	0.01	0.936
NHT * Species	0.65	0.421	1.33	0.254	0.16	0.691
T_{4wPost}	5.16	**0.026**	0.56	0.456	7.87	**0.007**
T_{4wPost} * Species	0.01	0.927	0.04	0.834	1.10	0.297
P_{4wPost}	0.03	0.866	0.56	0.455	1.02	0.317
P_{4wPost} * Species	0.37	0.544	0.14	0.712	0.01	0.919
P_{4wPost} * T_{4wPost}	<0.01	0.978	<0.01	0.999	0.03	0.860
P_{4wPost} * T_{4wPost} * Species	0.28	0.595	0.73	0.396	1.64	0.204

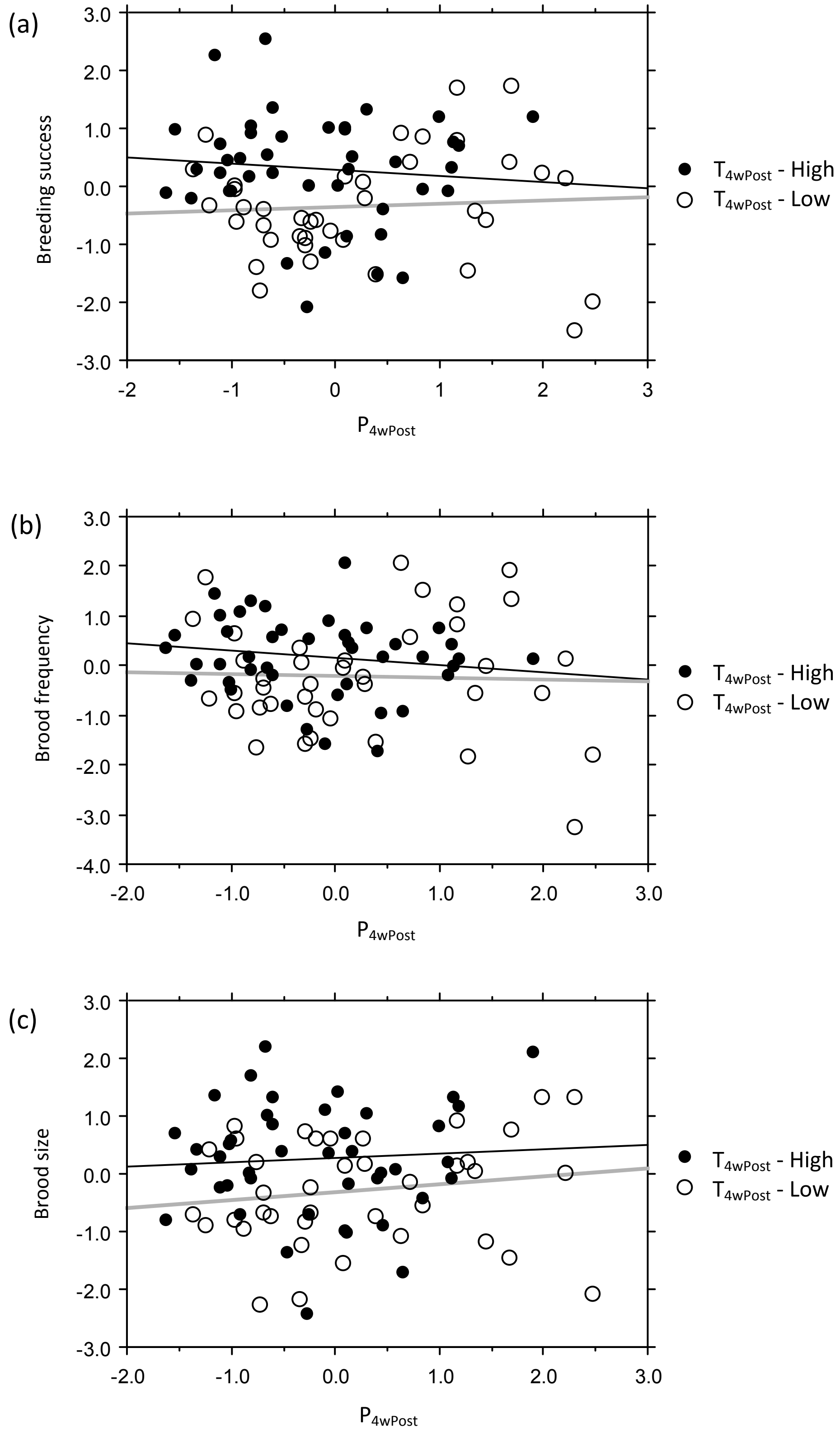

FIGURE D1. Scatterplots showing no interaction between temperature (T_{4wPost}) and precipitation (P_{4wPost}) during the four weeks period after hatching. Temperature given for above (high) and below (low) median values. All variables are standardised and detrended with NHT.

Appendix E: Species-specific effects on breeding success of explanatory variables. Hypotheses tested separately.

TABLE E1. Effects oncapercaillie and black grouse breeding success of the explanatory variables presented as partial β-coefficients (slopes, SEs) from multiple regressions within each hypothesis. The two species were treated as subjects in a repeated measures analysis of deviance via SAS (ver. 9.1) Proc Mixed, specifying “species nested in year” in the REPEATED statement and “variance components” as the covariance structure. Possible differences between species were tested by including interactions between them (categorical) and explanatory (continuous) variables. Statistically significant (one-tailed $p < 0.05$) effect sizes (β) are indicated with boldface. βs significantly different between species (two-tailed $p < 0.05$) are underlined.

Hypothesis	(n)	Explanatory variable	Capercaillie: Breeding success β (SE)	Capercaillie: Brood frequency β (SE)	Capercaillie: Brood size β (SE)	Black grouse: Breeding success β (SE)	Black grouse: Brood frequency β (SE)	Black grouse: Brood size β (SE)
Hen condition	(41)	NAO_w	**– 0.29** (0.15)	**– 0.33** (0.14)	– 0.11 (0.17)	**– 0.41** (0.16)	**– 0.39** (0.16)	– 0.09 (0.17)
		T_{8wPre}	**0.26** (0.15)	§) **0.34** (0.14)	– 0.03 (0.17)	– 0.03 (0.15)	§) – 0.08 (0.16)	0.02 (0.17)
		SnowFree	0.07 (0.15)	– 0.03 (0.15)	0.15 (0.18)	– 0.06 (0.16)	– 0.14 (0.17)	0.17 (0.17)
Chick weather	(41)	T_{4wPost}	**0.34** (0.17)	0.16 (0.17)	**0.42** (0.17)	**0.32** (0.18)	0.22 (0.18)	0.19 (0.19)
		P_{4wPost}	0.04 (0.15)	– 0.03 (0.16)	0.04 (0.16)	– 0.06 (0.15)	– 0.10 (0.16)	0.12 (0.16)
Chick food	(28)	Larvae	**0.39** (0.16)	**0.37** (0.17)	0.16 (0.20)	**0.57** (0.16)	**0.46** (0.17)	0.33 (0.20)
		$BB_{(t-1)}$	**0.50** (0.17)	**0.47** (0.17)	0.18 (0.21)	**0.42** (0.17)	0.14 (0.17)	**0.54** (0.21)
		$T_{JJA(t-12)}$	– 0.03 (0.18)	0.07 (0.19)	– 0.17 (0.23)	– 0.18 (0.18)	< 0.01 (0.19)	– 0.37 (0.23)
Predation	(38)	Voles	**0.42** (0.14)	**0.27** (0.15)	**0.28** (0.15)	0.22 (0.14)	– 0.09 (0.15)	**0.52** (0.15)
		Foxes	– 0.14 (0.15)	– 0.27 (0.16)	0.14 (0.16)	**– 0.51** (0.15)	**– 0.48** (0.16)	– 0.24 (0.16)
		$Grouse_{(t-23)}$	**– 0.32** (0.15)	– 0.20 (0.16)	**– 0.29** (0.15)	**– 0.37** (0.15)	**– 0.34** (0.16)	– 0.17 (0.16)
Demography	(40)	YoungHen	0.19 (0.19)	0.04 (0.19)	0.27 (0.20)	0.20 (0.17)	0.17 (0.17)	0.17 (0.18)
		DensHen	0.11 (0.17)	0.25 (0.17)	– 0.19 (0.17)	– 0.03 (0.17)	– 0.06 (0.17)	– 0.02 (0.17)
Demography - AR1	(39)	YoungHen	– 0.07 (0.19)	– 0.16 (0.20)	0.24 (0.19)	0.03 (0.18)	– 0.03 (0.18)	**0.31** (0.18)
		DensHen	0.24 (0.22)	0.36 (0.23)	– 0.15 (0.22)	– 0.09 (0.20)	– 0.13 (0.21)	0.04 (0.20)

§) The only β that is significantly different between species ($F_{4, 71} = 4.87$, $p = 0.03$, two-tailed).

Appendix F: Path analyses showing direct and indirect effect sizes of confounded explanatory variables in the Chick Food and Predation Hypotheses.

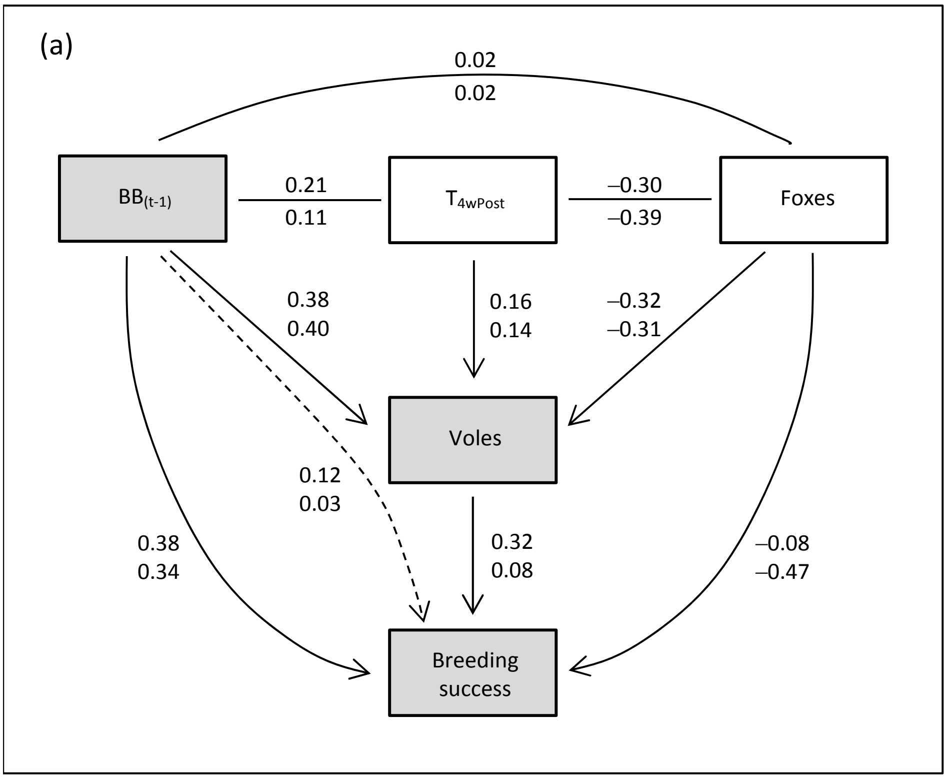

FIGURE F1. Path diagram showing direct and indirect (hatched lines) effect sizes for variables in the Predation Hypothesis (APH sub-hypothesis: Voles) and Chick Food Hypothesis (Plant Stress sub-hypothesis: $BB_{(t-1)}$). Effect sizes are shown for (a) breeding success, (b) brood frequency, and (c) brood size, with coefficients for capercaillie above black grouse. Detailed test statistics are shown in Table F1.

Figure F1 continues.

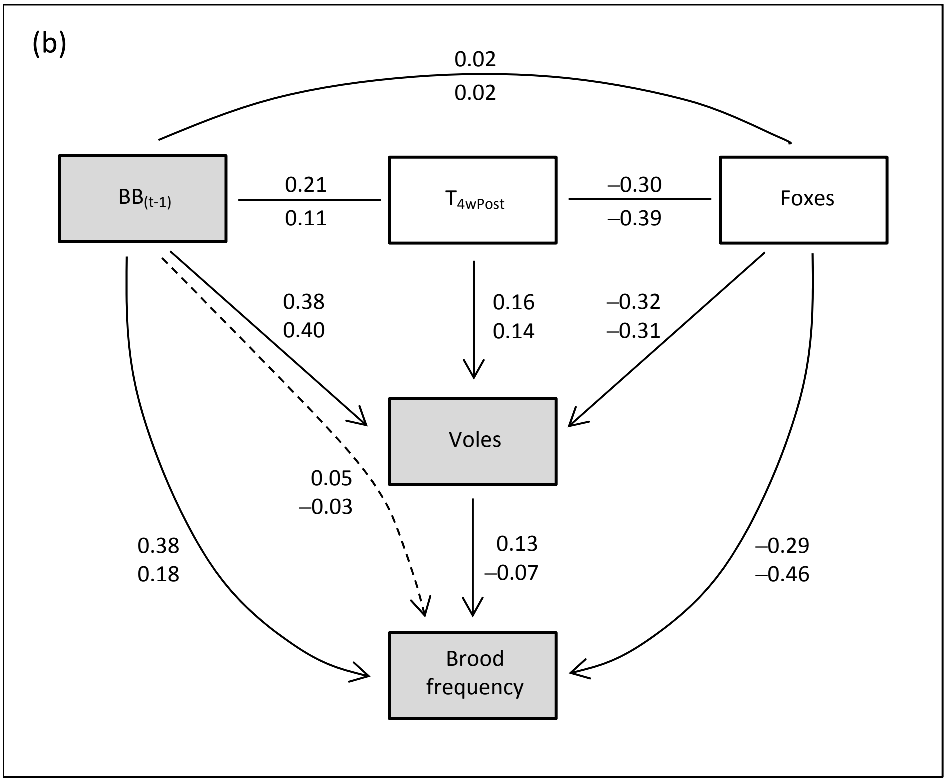

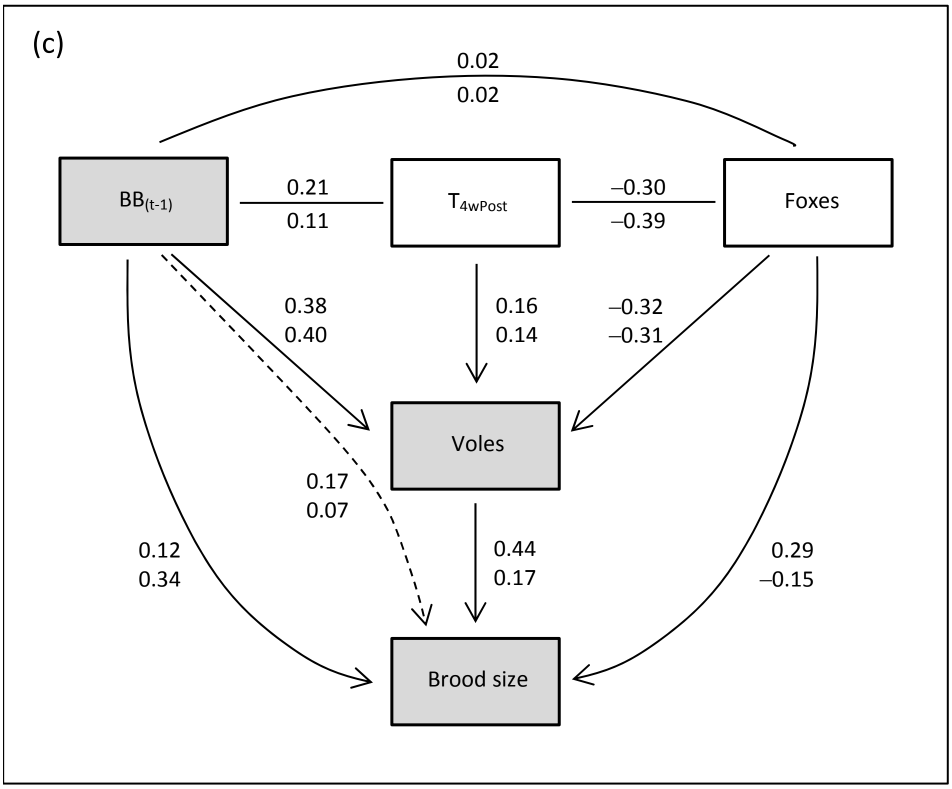

TABLE F1. Path coefficients for the effect of $BB_{(t-1)}$ on breeding success, directly and indirectly via Voles: (a) breeding success, (b) brood frequency and (c) brood size in capercaillie and black grouse. Indication of indirect effect is highlighted.

(a)

**BB(t-1) >> Voles >> Breeding success**	Capercaillie			Black Grouse			Combined	
	Path coeff.	SE	p_c [§]	Path coeff.	SE	p_c	χ^2	p_c
Direct effect	0.378	0.141	0.004	0.340	0.139	0.009	20.77	<0.001
Indirect effect	0.120	0.074	0.020	0.031	0.062	0.298	10.26	0.036
Total effect	0.498	0.159	0.001	0.371	0.151	0.008	23.41	0.001
Direct/indirect	3.2 ×			10.9 ×				
Indirect % of total	24 %			8 %				
**BB(t-1) >> Voles**	0.377	0.134	0.002	0.395	0.132	0.002	25.18	<0.001
**Voles >> Breeding success**	0.317	0.150	0.018	0.079	0.148	0.306	10.45	0.034

(b)

**$BB_{(t-1)}$ >> Voles >> Brood frequency**	Capercaillie			Black Grouse			Combined	
	Path coeff.	SE	p_c	Path coeff.	SE	p_c	χ^2	p_c
Direct effect	0.375	0.147	0.005	0.178	0.156	0.130	14.88	0.005
Indirect effect	0.049	0.065	0.206	−0.029	0.070	—	—	—
Total effect	0.423	0.163	0.004	0.149	0.172	0.194	14.14	0.007
Direct/indirect	7.7 ×			—				
Indirect % of total	11 %			—				
**Voles >> Brood frequency**	0.129	0.157	0.206	−0.073	0.167	---	—	—

(c)

**$BB_{(t-1)}$ >> Voles >> Brood size**	Capercaillie			Black Grouse			Combined	
	Path coeff.	SE	p_c	Path coeff.	SE	p	χ^2	p_c
Direct effect	0.119	0.153	0.215	0.336	0.155	0.013	11.81	0.019
Indirect effect	0.166	0.088	0.007	0.067	0.073	0.152	13.81	0.008
Total effect	0.285	0.180	0.056	0.403	0.171	0.010	15.05	0.005
Direct/indirect	0.72 ×			5.0 ×				
Indirect % of total	58 %			17 %				
**Voles >> Brood size**	0.440	0.164	0.004	0.169	0.166	0.153	15.01	0.005

§ p_c: Conditional probability.

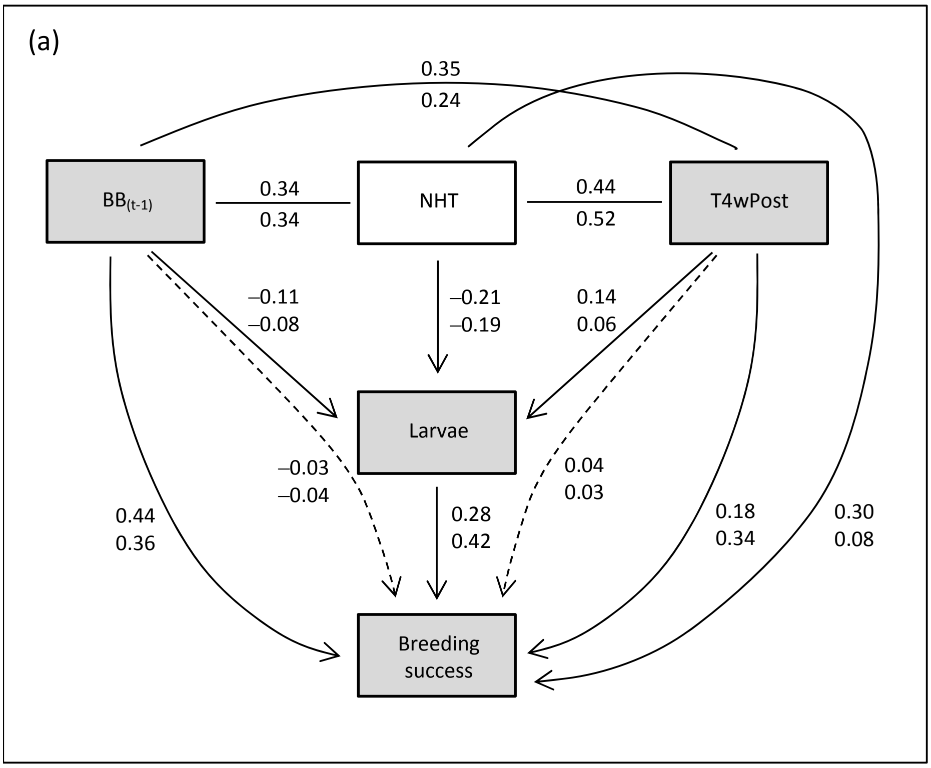

FIGURE F2. Path diagram showing direct and indirect (hatched lines) effect sizes for variables in the Chick Weather Hypothesis (T_{4wPost}) and Chick Food Hypothesis (Larvae and $BB_{(t-1)}$). Effect sizes are shown for (a) breeding success, (b) brood frequency, and (c) brood size, with coefficients for capercaillie above black grouse. Detailed test statistics are shown in Table F2 and F3.

Figure F2 continues.

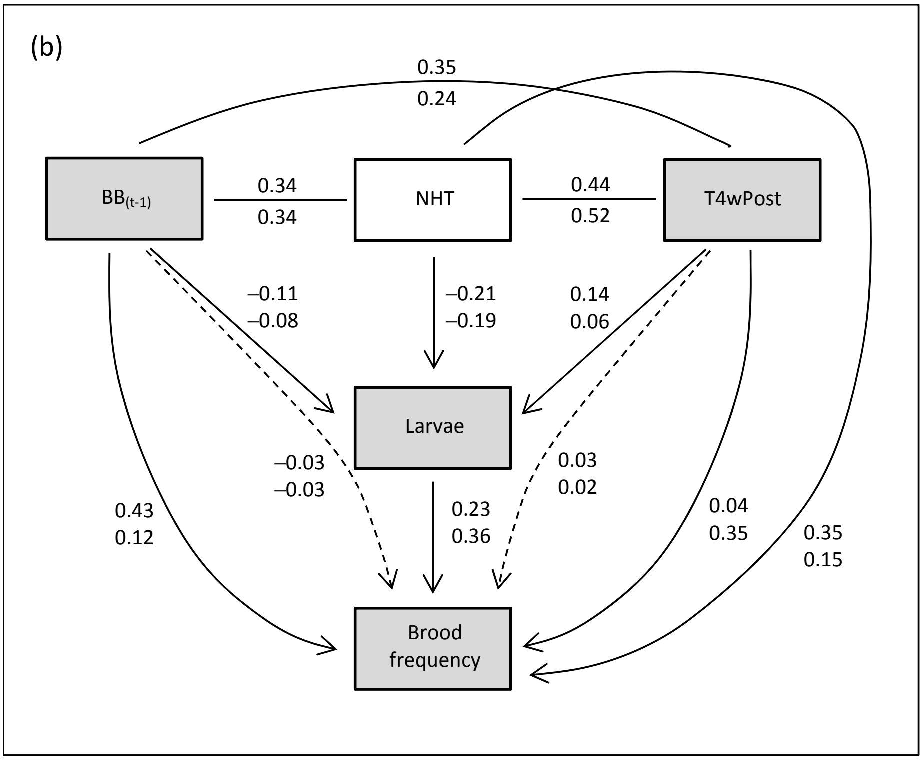

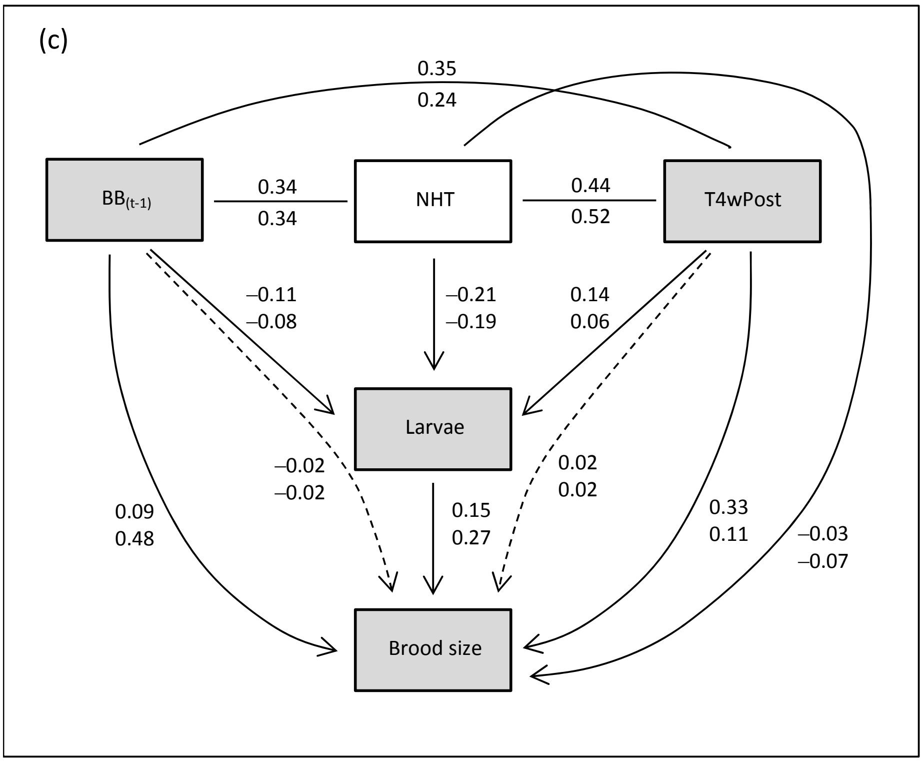

TABLE F2. Path coefficients for the effect of $BB_{(t-1)}$ on breeding success, directly and indirectly via Larvae: (a) breeding success, (b) brood frequency and (c) brood size in capercaillie and black grouse. No indication of indirect effects.

(a)

**$BB_{(t-1)}$ >> Larvae >> Breeding success**	Capercaillie			Black Grouse			Combined	
	Path coeff.	SE	p_c §	Path coeff.	SE	p_c	χ^2	p_c
Direct effect	0.440	0.146	0.001	0.357	0.150	0.009	22.50	<0.001
Indirect effect	−0.031	0.067	—	−0.035	0.091	—	—	—
Total effect	0.409	0.159	0.006	0.322	0.175	0.033	12.50	0.014
Direct/indirect	—			—				
Indirect % of total	—			—				
**$BB_{(t-1)}$ >> Larvae**	−0.110	0.205	—	−0.083	0.201	—	—	—
**Larvae >> Breeding success**	0.283	0.137	0.020	0.423	0.143	0.001	21.01	<0.001

(b)

**$BB_{(t-1)}$** >> Larvae >> **Brood frequency**	Capercaillie			Black Grouse			Combined	
	Path coeff.	SE	p_c	Path coeff.	SE	p_c	χ^2	p_c
Direct effect	0.434	0.159	0.003	0.122	0.169	0.229	14.50	0.006
Indirect effect	−0.025	0.058	—	−0.030	0.081	—	—	—
Total effect	0.409	0.170	0.009	0.092	0.185	0.309	11.79	0.019
Direct/indirect	—			—				
Indirect % of total	—			—				
**Larvae >> Brood frequency**	0.226	0.149	0.066	**0.363**	0.161	0.013	18.81	<0.001

(c)

**$BB_{(t-1)}$** >> Larvae >> **Brood size**	Capercaillie			Black Grouse			Combined	
	Path coeff.	SE	p_c	Path coeff.	SE	p_c	χ^2	p_c
Direct effect	0.088	0.195	0.344	0.478	0.175	0.003	13.69	0.008
Indirect effect	−0.016	0.052	—	−0.022	0.064	—	—	—
Total effect	0.072	0.204	0.380	0.456	0.188	0.007	11.83	0.019
Direct/indirect	—			—				
Indirect % of total	—			—				
**Larvae >> Brood size**	0.148	0.183	0.218	0.265	0.167	0.055	8.84	0.065

§ p_c: Conditional probability.

TABLE F3. Path coefficients for the effect of T_{4wPost} on breeding success, directly and indirectly via Larvae: (a) breeding success, (b) brood frequency and (c) brood size in capercaillie and black grouse. No indication of indirect effects.

(a)

T_{4wPost} >> Larvae >> Breeding success	Capercaillie			Black Grouse			Combined	
	Path coeff.	SE	p_c [§]	Path coeff.	SE	p_c	χ^2	p_c
Direct effect	0.183	0.153	0.114	0.344	0.165	0.020	12.19	0.016
Indirect effect	0.039	0.071	0.268	0.026	0.101	0.395	4.49	0.344
Total effect	0.222	0.167	0.092	0.370	0.193	0.030	12.32	0.015
Direct/indirect	4.7 ×			13.3 ×				
Indirect % of total	17 %			7 %				
**T_{4wPost} >> Larvae**	0.138	0.214	0.261	0.061	0.221	0.393	4.56	0.336
**Larvae >> Breeding success**	0.283	0.137	0.020	0.423	0.143	0.001	21.01	<0.001

(b)

T_{4wPost} >> Larvae >> Brood frequency	Capercaillie			Black Grouse			Combined	
	Path coeff.	SE	p_c	Path coeff.	SE	p_c	χ^2	p_c
Direct effect	0.036	0.167	0.410	0.351	0.185	0.026	9.05	0.060
Indirect effect	0.031	0.061	0.289	0.022	0.089	0.395	4.35	0.361
Total effect	0.067	0.173	0.349	0.373	0.205	0.034	8.86	0.065
Direct/indirect	1.2 ×			15.7 ×				
Indirect % of total	46 %			6 %				
**Larvae >> Brood frequency**	0.226	0.149	0.066	0.363	0.161	0.013	14.20	0.007

(c)

T_{4wPost} >> Larvae >> Brood size	Capercaillie			Black Grouse			Combined	
	Path coeff.	SE	p_c	Path coeff.	SE	p_c	χ^2	p_c
Direct effect	0.333	0.205	0.054	0.111	0.192	0.282	8.38	0.079
Indirect effect	0.021	0.056	0.345	0.016	0.071	0.402	3.95	0.413
Total effect	0.353	0.210	0.048	0.128	0.205	0.273	8.68	0.070
Direct/indirect	16.2 ×			6.9 ×				
Indirect % of total	6 %			13 %				
**Larvae >> Brood size**	0.148	0.183	0.218	0.265	0.167	0.055	8.84	0.0652

§ p_c: Conditional probability.
